# Metabolic signature of EAT-Lancet diet in relation to risk of frailty: a prospective cohort study

**DOI:** 10.1038/s41538-025-00619-0

**Published:** 2025-11-26

**Authors:** Jing Guo, Guangning Kou, Yongjian Zhu, Rui Liang, Hua Ye, Xiangying Suo, Yacong Bo

**Affiliations:** 1 Department of Nutrition and Food Hygiene, College of Public Health, Zhengzhou University, Zhengzhou, China; 2 Department of Kinesiology, School of Physical Education (Main Campus), Zhengzhou University, Zhengzhou, China; 3 Centre for Nutritional Ecology and Centre for Sport Nutrition and Health, Zhengzhou University, Zhengzhou, China; 4 Department of Cardiology, The First Affiliated Hospital of Zhengzhou University, Zhengzhou, China; 5 Department of Nutrition, the First Affiliated Hospital of Zhengzhou University, Zhengzhou, China; 6 Henan Key Laboratory of Tumor Epidemiology, Zhengzhou, China; 7 Henan International Joint Laboratory of Tumor Biomarkers and Molecular Imaging, Zhengzhou, China

**Keywords:** Metabolomics, Fatigue

## Abstract

The EAT-Lancet Commission proposed a planetary health diet in 2019, yet its association with frailty remains unclear. This study aimed to examine the relationships between adherence to the EAT-Lancet diet, metabolomic profiles, and frailty risk among older adults. We included 44,465 participants from the UK Biobank, with a mean age of 55.88 years. Frailty was defined based on five physical criteria. Elastic net regression identified a 20-metabolite signature associated with the EAT-Lancet diet. Cox models showed that higher diet adherence was significantly associated with reduced frailty risk (HR: 0.51, 95% CI: 0.40 to 0.64). Each standard deviation increase in the metabolic signature was also associated with lower frailty risk (HR: 0.69, 95% CI: 0.63 to 0.75). Mediation analysis indicated that metabolites mediated 9.88% of the diet-frailty association. These findings suggest that greater adherence to the EAT-Lancet diet may lower frailty risk, partly mediated through specific metabolic pathways.

## Introduction

Frailty is a complex, multifaceted syndrome characterized by diminished physiological reserves and reduced resistance to stressors, posing a significant health challenge for aging populations globally^1,2^. Previous studies have suggested that frailty is associated with an increased risk of adverse outcomes such as falls^3^, fractures^4^, disability^5^, and mortality^6^. Individuals suffering from frailty also endure significant personal burdens, such as diminished quality of life and increased risk of social isolation^7,8^. A recent meta-analysis suggested that the prevalence of physical frailty is approximately 12% among adults aged 50 and older^9^. Frailty is undeniably one of the most pressing global public health challenges of the coming century. The rapid growth of the aging population has led to a corresponding increase in the number of frail older adults, further straining healthcare systems worldwide^10,11^. Consequently, identifying and managing modifiable risk factors for frailty are critical for promoting the health and independence of older adults.

Recent evidence has highlighted the role of dietary factors in the development and progression of age-related disorders. Several studies have identified strong positive associations between various healthy dietary patterns and frailty^12–14^. However, a healthy diet should be considered not only for health benefits but also for its environmental impact, as food preparation is a major driver of global climate change. In this context, the EAT-Lancet Commission introduced a global reference diet specifically designed to promote both human health and environmental sustainability^15^. This dietary pattern, termed the EAT-Lancet diet, prioritizes vegetables, fruits, whole grains, legumes, nuts, and unsaturated oils, while recommending moderate consumption of seafood, poultry, and dairy, and limiting meat, added sugars, refined grains, and starchy vegetables^16^. Although previous research has examined the long-term health effects of EAT-Lancet diet, such as its impact on cardiovascular disease (CVD)^17^, type 2 diabetes^18^, metabolic dysfunction-associated steatotic liver disease^19^, and mortality^20^, its association with the risk of frailty remains unexplored.

The investigation of metabolic markers is essential for understanding the potential role of metabolic status in aging-related diseases and provides a scientific foundation for targeted prevention strategies. Metabolomics, which involves comprehensive profiling of metabolites in biological matrices, provides a systems-level perspective on biological responses to environmental and behavioral influences^21^. Epidemiological studies have demonstrated associations of different lifestyles with several chronic diseases, such as type 2 diabetes and CVD, using metabolomics^22,23^. However, the impact of the EAT-Lancet diet on frailty risk via metabolic status remains unclear and warrants further exploration. Metabolomics can contribute to this understanding in several ways. First, it can elucidate the biological mechanisms through which dietary changes influence frailty, including the role of specific metabolites in mediating this relationship. Second, it can identify early metabolic biomarkers that may predict frailty risk before clinical symptoms emerge. Additionally, given the diverse components of the EAT-Lancet diet, metabolomics can help disentangle the specific contributions of individual dietary elements to frailty risk. Furthermore, integrating metabolomics into the EAT-Lancet diet studies and identifying metabolic signatures associated with this diet could provide new insights into the biological mechanisms linking the relationship between EAT-Lancet diet and risk of frailty.

Therefore, we aimed to investigate the associations of adherence to EAT-Lancet diet with risk of frailty in the UK Biobank, with further investigation into the potential mediating role of metabolic signatures.

## Results

### Demographic characteristics

A total of 44,465 participants were included in this study, of whom 23,098 (51.9%) were female, with a mean age of 55.88 ± 7.56 years. During a median follow-up of 9.10 years, 1,120 incident cases of frailty were identified. Participants who developed frailty were more likely to be female, unemployed, and have a higher BMI and lower education level. In addition, they were more likely to have adverse factors such as low PA levels, a history of drinking and smoking (Table 1). We also compared the baseline characteristics of the participants according to the categories of EAT-Lancet score (Supplementary Table 1). Participants with a higher EAT-Lancet score were more likely to be female, have a lower BMI and higher education level, and have healthier habits, such as high levels of PA and never smoking.

**Table 1:** Baseline characteristics of the participants included in this study

Characteristics	Overall (*N* = 44465)	Non-frailty	Frailty
		*N* = 43345	*N* = 1120
Female, *n* (%)	23098 (51.9)	22467 (51.8)	631 (56.3)
Age, mean (SD), *y*	55.88 ± 7.56	55.82 ± 7.55	57.90 ± 7.65
BMI, mean (SD)	26.52 ± 4.28	26.42 ± 4.16	30.49 ± 6.30
Race, white, *n* (%)	43139 (97.0)	42066 (97.0)	1073 (95.8)
Townsend Deprivation index, mean (SD)	−1.94 ± 2.69	−1.96 ± 2.68	−1.23 ± 3.03
Education, *n* (%)			
College or University	22155 (49.8)	21746 (50.2)	409 (36.5)
Other	22310 (50.2)	21599 (49.8)	711 (63.5)
Employment, *n* (%)			
No	14688 (33.0)	14142 (32.6)	546 (48.8)
Yes	29777 (67.0)	29203 (67.4)	574 (51.2)
Physical activity, *n* (%)			
Low	8193 (18.4)	7796 (18.0)	397 (35.4)
Moderate	18887 (42.5)	18433 (42.5)	454 (40.5)
High	17385 (39.1)	17116 (39.5)	269 (24.0)
Drinking, *n* (%)			
Current	42405 (95.4)	41414 (95.5)	991 (88.5)
Previous	978 (2.2)	908 (2.1)	70 (6.2)
Never	1082 (2.4)	1023 (2.4)	59 (5.3)
Smoke, *n* (%)			
Current	2565 (5.8)	2462 (5.7)	103 (9.2)
Previous	14989 (33.7)	14554 (33.6)	435 (38.8)
Never	26911 (60.5)	26329 (60.7)	582 (52.0)

*BMI* body mass index.

### Association of EAT-Lancet diet with the risk of frailty

**Fig. 1:**
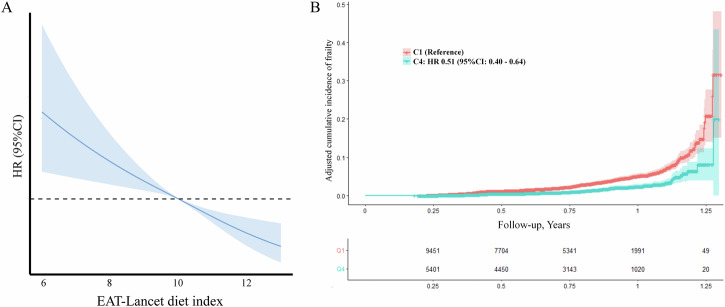
Effect of EAT-Lancet diet on the risk of frailty. **A** Restricted cubic splines for the association of EAT-Lancet diet score with risk of frailty. **B** Cumulative frailty incidences for Category 1 (≤9) and Category 4 (≥11) of EAT-Lancet score. All HRs and 95% CIs were estimated using Cox proportional hazard models with adjustment for sex, age, PA, education, employment, smoking, drinking, Townsend deprivation index, energy intake, CVD, and cancer. PA, physical activity; CVD, cardiovascular disease.

Compared with the lowest quartile of EAT-Lancet diet score, the HR (95% CI) of participants in the highest category was 0.47 (0.37–0.59). After adjusting for a series of covariates, the relationship remained consistent (HR 0.51, 95% CI, 0.40–0.64), and the association showed significant linearity (*P-*_nonlinear_ = 0.556, Fig. 1A and Table 2). In response to specific components of individual frailty (Table 3), we found that the EAT-Lancet diet score was inversely associated with symptoms of frailty including exhaustion (HR: 0.71, 95% CI: 0.63 to 0.81), low physical activity (HR: 0.58, 95% CI: 0.51–0.67), slow walking speed (HR: 0.58, 95% CI: 0.49–0.68), weak hand grip (HR: 0.90, 95% CI: 0.83–0.98) and weight loss (HR: 0.86, 95% CI: 0.80–0.94). The individual components of the EAT-Lancet diet show that following the guidelines’ recommendations to increase vegetables (HR: 0.79, 95% CI: 0.70–0.89) and fruits (HR: 0.68, 95% CI: 0.60–0.78) intake and consume dairy products (HR: 0.79, 95% CI: 0.65– 0.95) in moderation is significantly associated with a lower risk of frailty. At the same time, reducing the intake of beef/mutton/pork (red meat) (HR: 0.86, 95% CI: 0.76 to 0.97) or added fats (HR: 0.66, 95% CI: 0.46–0.94) is significantly associated with a lower risk of frailty. Conversely, high added sugar (HR: 1.55, 95% CI: 1.05–2.29) intake significantly increases the risk of frailty (Supplementary Table 2). Additionally, we performed a cumulative risk analysis to validate the cumulative risk of frailty over time, which showed that participants with a higher EAT-Lancet diet score had a lower cumulative incidence of frailty compared to those with lower EAT-Lancet diet score (Fig. 1B).

**Table 2:** Association of the EAT-Lancet diet score and the risk of frailty

	Categories of the EAT-Lancet diet score			
	≤9	=10	=11	≥12
Cases/Person-Years	335/87897	375/132397	324/134296	86/50229
Model 1^a^	Ref	0.75 (0.64–0.87)	0.63 (0.54–0.74)	0.47 (0.37–0.59)
Model 2^b^	Ref	0.70 (0.60–0.81)	0.56 (0.48–0.65)	0.41 (0.32–0.51)
Model 3^c^	Ref	0.75 (0.64–0.87)	0.65 (0.55–0.76)	0.51 (0.40–0.64)

*Ref* reference.

^a^Original model without adjusting for any variables.

^b^Adjusted for age, sex, and energy.

^c^Adjusted for age, sex, energy, PA, Townsend deprivation index, education, employment, smoking, drinking, CVD and cancer.

**Table 3:** Association of the EAT-Lancet diet score and the risk of frailty

	Categories of the EAT-Lancet diet score			
	≤9	=10	=11	≥12
Exhaustion				
Cases/Person-years	529/79304	748/121842	724/124348	221/46673
Model 1^a^	Ref	0.93 (0.83−1.04)	0.88 (0.78−0.98)	0.74 (0.64−0.87)
Model 2^b^	Ref	0.90 (0.81−1.01)	0.83 (0.74−0.93)	0.68 (0.58−0.80)
Model 3^c^	Ref	0.89 (0.82−0.98)	0.83 (0.75−0.90)	0.71 (0.63−0.81)
Low physical activity				
Cases/Person-years	580/78888	730/122565	620/125920	185/47760
Model 1^a^	Ref	0.82 (0.73−0.91)	0.67 (0.60−0.76)	0.55 (0.46−0.64)
Model 2^b^	Ref	0.78 (0.70−0.88)	0.63 (0.56−0.70)	0.51 (0.43−0.60)
Model 3^c^	Ref	0.80 (0.73−0.88)	0.70 (0.63−0.77)	0.58 (0.51−0.67)
Slow walking speed				
Cases/Person-years	432/85281	490/129114	474/131555	149/49597
Model 1^a^	Ref	0.76 (0.66−0.86)	0.72 (0.63−0.82)	0.63 (0.52−0.76)
Model 2^b^	Ref	0.71 (0.62−0.81)	0.63 (0.55−0.71)	0.55 (0.46−0.67)
Model 3^c^	Ref	0.79 (0.71−0.87)	0.70 (0.63−0.79)	0.58 (0.49−0.68)
Weak hand grip				
Cases/Person-years	1072/75759	1610/113870	1634/115553	569/43218
Model 1^a^	Ref	1.01 (0.93−1.09)	1.01 (0.93−1.09)	0.99 (0.90−1.10)
Model 2^b^	Ref	0.93 (0.86−1.01)	0.86 (0.79−0.93)	0.85 (0.77−0.94)
Model 3^c^	Ref	0.94 (0.89−1.00)	0.87 (0.82−0.92)	0.90 (0.83−0.98)
Weight loss				
Cases/Person-years	1532/75481	2098/113272	2084/115838	741/43700
Model 1^a^	Ref	0.92 (0.86−0.98)	0.89 (0.83−0.95)	0.86 (0.79−0.94)
Model 2^b^	Ref	0.90 (0.84−0.96)	0.86 (0.80−0.92)	0.84 (0.77−0.92)
Model 3^c^	Ref	0.91 (0.86−0.96)	0.88 (0.83−0.93)	0.86 (0.80−0.94)

*Ref* reference.

^a^Original model without adjusting for any variables.

^b^Adjusted for age, sex and energy.

^c^Adjusted for age, sex, energy, PA, townsend deprivation index, education, employment, smoking, drinking, CVD and cancer.

### Stratified and sensitivity and analyses

In stratified analyses for frailty, the results showed that the relationship between EAT-Lancet diet and frailty was consistent among the different subgroups of age, smoking status, drinking status, PA, and BMI (Table 4), yet females were at a lower risk of developing frailty than male in the sex stratification. In further analysis, we continued to observe a negative correlation between the EAT-Lancet diet score and the risk of frailty. This association remained robust across multiple sensitivity analyses, including excluding participants who developed frailty within 2 years of baseline; excluding participants with cardiovascular disease or cancer at baseline; further adjusting for BMI, depressive status, and polypharmacy to control for potential confounders; and calculating diet scores using the average of all available dietary assessments. Collectively, these results suggest that residual confounding due to mental health or overall disease burden did not undermine the main findings (Supplementary Table 3).

**Table 4:** Subgroup analysis of EAT-Lancet diet score with incident frailty

	Categories of the EAT-Lancet diet score				
	≤9	=10	=11	≥12	*P*-interation
Sex					<0.001
Male	Ref	0.78 (0.63−0.97)	0.64 (0.51−0.81)	0.53 (0.35−0.79)	
Female	Ref	0.72 (0.59−0.89)	0.65 (0.53−0.80)	0.49 (0.36−0.67)	
Age					0.433
<65	Ref	0.77 (0.65−0.90)	0.63 (0.52−0.75)	0.52 (0.40−0.68)	
≥65	Ref	0.67 (0.47−0.94)	0.71 (0.51−0.99)	0.45 (0.26−0.78)	
Smoking					0.078
Yes	Ref	0.80 (0.64−0.98)	0.74 (0.60−0.93)	0.47 (0.32−0.68)	
No	Ref	0.70 (0.57−0.86)	0.56 (0.45−0.70)	0.52 (0.38−0.72)	
Drinking					0.737
Yes	Ref	0.78 (0.67−0.90)	0.66 (0.57−0.78)	0.51 (0.40−0.66)	
No	Ref	0.45 (0.23−0.88)	0.48 (0.24−0.95)	0.42 (0.16−1.09)	
Physical activity					0.097
Low	Ref	0.70 (0.54−0.89)	0.73 (0.56−0.94)	0.67 (0.45−0.99)	
Moderate/High	Ref	0.77 (0.64−0.93)	0.60 (0.50−0.73)	0.43 (0.32−0.58)	
Body mass index					0.601
<25	Ref	0.73 (0.50−1.05)	0.63 (0.43−0.91)	0.66 (0.41−1.04)	
≥25	Ref	0.78 (0.66−0.91)	0.69 (0.58−0.82)	0.52 (0.39−0.70)	

*Ref* reference.

Adjusted for age, sex, energy, PA, Townsend deprivation index, education, employment, smoking, drinking, CVD and cancer.

### Variation of the metabolites in response to EAT-Lancet diet

**Fig. 2:**
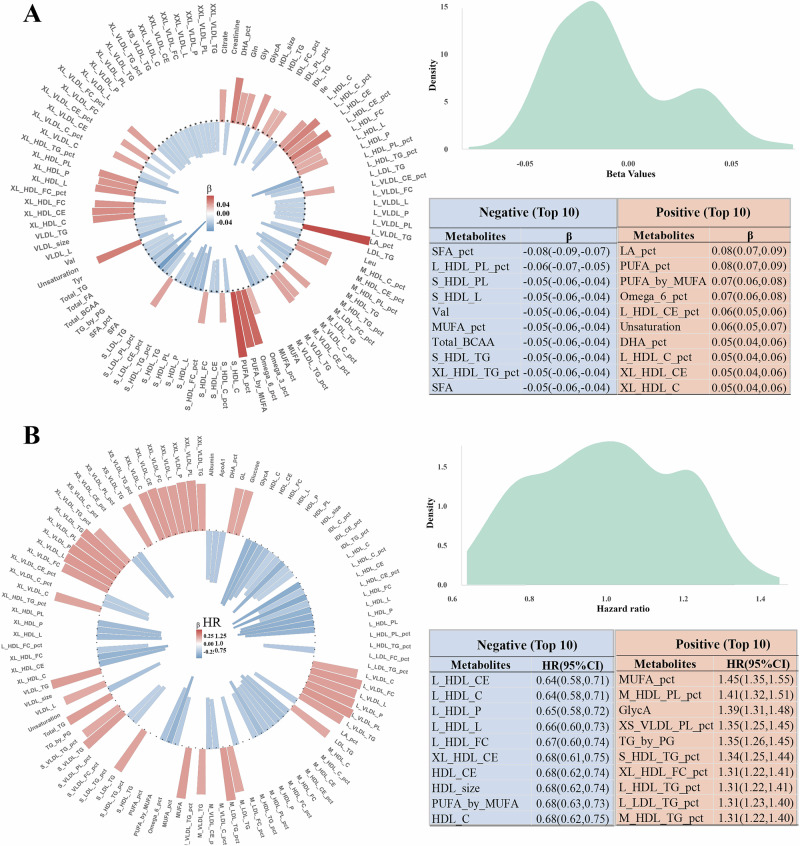
Association of metabolites with EAT-Lancet diet and frailty. **A** Association of EAT-Lancet diet with metabolites. **B** Association of frailty with metabolites. HRs and 95% CIs were estimated using Cox proportional hazard models, and β was calculated using linear regression models, all adjusted for sex, age, PA, education, employment, smoking, drinking, Townsend deprivation index, energy intake, CVD, and cancer. *PA* physical activity, CVD cardiovascular disease.

We conducted elastic net regression analyses on 251 metabolites to identify the metabolic signatures of the EAT-Lancet diet. The results showed a combination of 211 metabolites associated with the EAT-Lancet diet (Fig. 2A and Supplementary Table 4). The results showed that linoleic acid percentage (LA_pct) and polyunsaturated fatty acids percentage (PUFA_pct) were the most strongly positively associated with the EAT-Lancet diet. However, saturated fatty acids percentage (SFA_pct) showed the strongest negative association with the EAT-Lancet diet. The metabolic signatures were further calculated as the weighted sum of the selected metabolites with weights equal to the coefficients from the elastic net regression. Metabolites that constituted the metabolic signature encompassed a range of metabolic categories, such as amino acids, fatty acids, lipoprotein particle sizes, lipoprotein subclasses and glycolysis-related metabolites. Notably, amino acids and fatty acids predominated in the metabolic signatures.

### Associations of metabolic signatures with risk of incident frailty

**Fig. 3:**
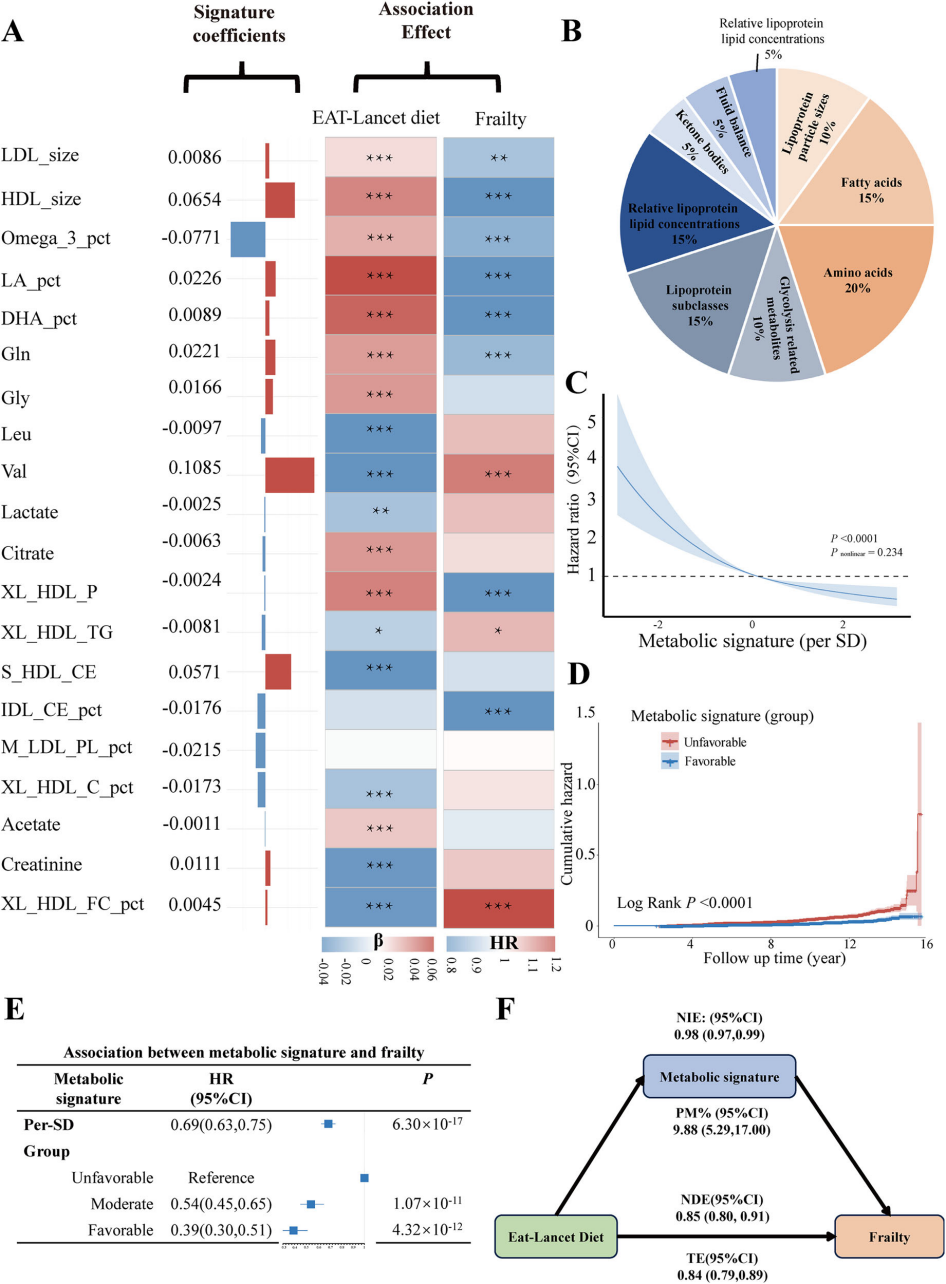
Metabolic signatures and risk of frailty incidents. **A** Metabolic signatures weighted in EAT-Lancet diet and frailty. **B** Category of metabolic signatures. **C** Restricted cubic splines for the association of metabolic signatures with risk of frailty. **D** Cumulative frailty incidences for Croup 1 (unfavorable) and Group 3 (favorable) of metabolic signatures. **E** Association between metabolic signatures and frailty. **F** Mediation analysis with metabolites as potential mediators of the association between EAT-Lancet score and frailty risk. All HRs and 95% CIs were estimated using Cox proportional hazard models with adjustment for sex, age, PA, education, employment, smoking, drinking, Townsend deprivation index, energy intake, CVD, and cancer. PA physical activity, CVD cardiovascular disease.

Metabolic signatures associated with adherence to the EAT-Lancet diet were linked to a lower risk of frailty. Analysis of individual metabolites and incident frailty identified 180 metabolites significantly associated with frailty (Fig. 2B and Supplementary Table 4). Additionally, strong associations were observed between the individual metabolites within the metabolic signatures and frailty. Specifically, 11 metabolites were identified as significant. For example, a higher percentage of linoleic acid and docosahexaenoic acid to fatty acids was associated with a lower risk of frailty, whereas a higher percentage of free cholesterol to total lipids in very large HDL (XL_HDL_FC_pct) was associated with higher frailty risk (Fig. 3A). Composite signatures related to the EAT-Lancet diet and frailty risk were characterized by weight distributions and categorical profiles, including amino acids, fatty acids, and lipoprotein subclasses (Fig. 3B)

Restricted cubic spline analysis demonstrated a significant inverse trend between increasing levels of the metabolic signatures related to the EAT-Lancet diet and frailty risk (*P*_for overall_ < 0.001, *P*_for nonlinear_ = 0.234, Fig. 3C). The cumulative risk curve further confirmed this association, indicating that individuals with a higher favorable metabolite signatures exhibited an approximately 61% reduced frailty risk compared to those with unfavorable levels (HR: 0.39, 95% CI: 0.30−0.51) (Fig. 3D, E).

### Mediating roles of metabolites

The metabolites partly mediated the association between the EAT-Lancet diet and frailty risk, as shown in Fig. 3F. The analysis results showed that the total effect (TE) was 0.19. Further decomposition of the effects revealed that the natural direct effect (NDE) was 0.85 (95% CI: 0.80−0.91), while the natural indirect effect (NIE) was 0.98 (95% CI: 0.97−0.99). The NIE accounted for 9.88% of the total effect, suggesting a statistically significant mediating role of metabolites in the relationship between the EAT-Lancet diet and frailty risk.

## Discussion

In this study, we are the first reported that greater adherence to the EAT-Lancet diet is linked to a lower risk of frailty. Additionally, we identified a panel of metabolites associated with the EAT-Lancet diet and developed a corresponding metabolic signature. This signature comprised 20 metabolites related to the EAT-Lancet diet and was further associated with a reduced risk of incident frailty. Mediation analysis revealed that metabolic signatures accounted for 9.88% of the association between adherence to the EAT-Lancet diet and frailty.

Although previous studies have investigated the relationship between different dietary patterns and the risk of frailty, the methods used to define dietary patterns have varied. A “prudent” pattern, characterized by high consumption of olive oil and vegetables, was found to have a negative dose-response relationship with the risk of frailty. In contrast, a “Westernized” dietary pattern, with high intake of refined bread and red meat, and low consumption of fruits and vegetables, was associated with an increased risk of specific frailty phenotypes, such as slow gait speed and weight loss^24^. The Nurses’ Health Study (NHS) demonstrated that the Mediterranean diet could reduce the risk of frailty in an older population^25^. Another study based on the same cohort found that adherence to the alternate Mediterranean diet (AMED), the alternate Healthy Eating Index-2010 (AHEI-2010), and the Dietary Approaches to Stop Hypertension (DASH) diet similarly reduced the risk of frailty in older women^26^. Additionally, research from the National Health and Nutrition Examination Survey (NHANES) also indicated a relationship between diet quality scores and frailty risk across a wide age range^27^.

However, these dietary patterns partially overlap with those proposed by the EAT-Lancet Commission, most do not fully address the inclusion of unhealthy food options from both plant and animal sources. Unlike previous plant-based dietary patterns that exclude all animal-derived foods, the EAT-Lancet approach incorporates not only plant-based foods but also healthy animal-derived options such as dairy and fish. This flexibility may make it more practical and appealing dietary option. A healthy diet should provide an appropriate calorie intake, emphasizing a variety of plant-based foods, moderate amounts of animal foods, the replacement of saturated fats with unsaturated fats, and the reduction of refined grains, highly processed foods, and added sugars^15^. While there are varying interpretations of plant-based diets, it is important to recognize the health benefits of gradually increasing the intake of plant-based foods and decreasing animal-based foods, rather than adopting a strictly vegan diet that eliminates all animal products.

Component analysis of the EAT-Lancet diet revealed distinct associations between specific food groups and frailty risk. Higher consumption of vegetables and fruits—consistent with the guideline’s emphasis on plant-based foods—demonstrated a significant inverse association with frailty incidence. Similarly, adherence to recommended moderate dairy intake was linked to reduced frailty risk. Conversely, lower consumption of red meat and added fats aligned with guideline targets and showed protective effects against frailty. Notably, exceeding the EAT-Lancet’s added sugar threshold exhibited a strong positive association with frailty development. Therefore, following the EAT-Lancet dietary priorities, specifically including increasing vegetable and fruit intake, moderate dairy intake, reducing red meat and added fats, and strictly limiting added sugars, is recommended to reduce the risk of frailty. In subgroup analyses, the protective association between adherence to the EAT-Lancet diet and incident frailty was significantly stronger in females than in males (*P*-interaction *<* 0.001). Several factors may contribute to this sex-based difference. First, estrogen has been shown to enhance mitochondrial efficiency and modulate anti-inflammatory pathways, which may synergize with the plant-based components of the EAT-Lancet diet, such as polyphenols and omega-3 polyunsaturated fatty acids (ω-3 PUFAs)^28,29^. Second, women generally exhibit higher adherence to health-promoting dietary patterns, which could strengthen the observed association^30^. Lastly, social and behavioral roles—such as greater involvement in food preparation—may also influence dietary engagement and consistency among women.

In this study, we developed a metabolomic signature score reflecting adherence to the EAT-Lancet diet. We then simultaneously examined the relationship between this diet and its associated metabolomic signature score with frailty. As expected, our findings suggest that the metabolomic signature score linked to the EAT-Lancet diet may reduce the likelihood of developing frailty over time. Consistent with prior research, our study reinforces the connection between the EAT-Lancet diet and a broad spectrum of metabolites^31^, Additionally, the fact that many of these metabolites are also correlated with frailty incidence aligns with findings from an earlier prospective study^32^. Further investigation is required to elucidate the precise biological mechanisms underlying the metabolic alterations induced by the EAT-Lancet diet.

The metabolic signature incorporated 20 metabolites, including lipoproteins, amino acids, and fatty acids. Although our findings did not directly assess functional outcomes, prior research suggests that ω-3 PUFAs may support physical function—an essential component of frailty—through several biological mechanisms^33,34^. For instance, ω-3 PUFAs have been associated with reduced inflammation, which may mitigate age-related declines in muscle mass and strength, both key features of sarcopenia and frailty^35^. Additionally, ω-3 PUFAs are incorporated into muscle cell membranes, potentially supporting protein synthesis and neuromuscular integrity^36,37^. Beyond their anti-inflammatory effects, ω-3 PUFAs may also influence oxidative stress pathways^38,39^. Some studies have shown that supplementation can enhance the body’s antioxidant defenses—for example, by upregulating Nrf2-mediated detoxification pathways and increasing superoxide dismutase (SOD) activity, while reducing markers of lipid peroxidation such as TBARS^40,41^. However, we acknowledge the well-documented susceptibility of ω-3 PUFAs to oxidation. In this context, tocopherol (vitamin E), often co-present in dietary sources of ω-3 PUFAs, plays a key protective role by preventing lipid peroxidation during storage and metabolism^42^. Frailty in older adults is significantly associated with gut microbiota dysbiosis, characterized by reduced diversity, decreased abundance of specific beneficial bacteria, and increased abundance of potentially harmful bacteria^43^. Notably, glutamine has been demonstrated to promote enterocyte proliferation, modulate tight junction proteins, suppress pro-inflammatory signaling pathways, and provide protection against cell death and cellular stress under both normal and pathological conditions^44,45^. Furthermore, as a conditionally essential amino acid, glutamine plays a particularly important role in regulating muscle protein synthesis^46^. Given that muscle atrophy is a key contributor to frailty, linoleic acid (LA)—a major component of cell membrane phospholipids—may support muscle function by maintaining membrane fluidity. Although LA is a precursor of PGE2, studies in healthy adults indicate that dietary intake within physiological ranges does not increase systemic inflammatory markers^47,48^. Moreover, epidemiological evidence suggests that LA intake between 11.4 and 19.2 g/day is associated with a lower risk of frailty in middle-aged and older adults^49^. Notably, this essential fatty acid can be obtained through daily dietary sources such as legumes, vegetables, and fish. Therefore, the metabolic signature variations observed in the present study may partially originate from the long-term effects of adhering to the EAT-Lancet dietary pattern, thus supporting our hypothesis that the diet reduces the risk of frailty by influencing metabolism.

We observed a modest mediation proportion for the relationship between the EAT-Lancet diet and frailty risk (9.88%). This could be due to the included metabolic signatures, which reflect processes like amino acid and lipid metabolism, likely only partially capturing the complex biological pathways linking diet to frailty. Emerging evidence implicates significant roles for alternative mechanisms—particularly involving gut microbiome modulation, immune function, or endocrine signaling—which were not assessed by our metabolomics platform. Furthermore, the untargeted NMR approach employed, while robust, has lower sensitivity for key metabolite classes potentially critical to mediation (e.g., short-chain fatty acids, bile acids, microbiota-derived compounds) compared to mass spectrometry. Finally, the protective effects of the EAT-Lancet diet, integrating nutrient adequacy and sustainability, may also operate through behavioral or psychosocial pathways (e.g., meal regularity, dietary variety) not reflected in circulating metabolomic profiles. Furthermore, A key candidate is the gut microbiome, which is known to mediate the health effects of plant-based diets^12^. The EAT-Lancet dietary pattern, rich in fiber and polyphenols, promotes the growth of beneficial gut microbes that ferment dietary substrates into short-chain fatty acids (SCFAs), such as acetate, propionate, and butyrate^50–52^. These microbial metabolites exert anti-inflammatory effects, enhance gut barrier integrity, modulate immune function, and influence energy homeostasis—all of which are relevant to the pathophysiology of frailty^53^. Furthermore, gut microbiota metabolize polyphenols into bioactive compounds (e.g., urolithins) with anti-inflammatory and antioxidant properties^54^. However, many of these microbially derived metabolites are not detectable using standard plasma NMR platforms due to their low concentrations, rapid metabolism, or requirement for specialized analytical techniques. Therefore, the gut microbiome likely represents a major unmeasured mediator of the diet-frailty association. Future studies should integrate high-resolution microbiome profiling with mass spectrometry-based metabolomics to more comprehensively capture the complex host–microbe–diet interactions underlying frailty risk.

This study has several important strengths. To the best of our knowledge, our study is one of the first to elucidate the metabolic mechanisms underlying the reduction in frailty risk associated with the EAT-Lancet diet pattern. The strengths of our study include a large sample size, a long follow-up period, and sensitivity analyses for potential confounders, all of which minimize bias and enhance the robustness of the findings. Additionally, we applied elastic net regression modeling for the first time to develop a metabolic signature representing adherence to the EAT-Lancet diet. Identifying 20 metabolites associated with this diet and constructing a corresponding metabolic signature may provide novel biomarkers or screening tools for evaluating frailty risk. However, there are also some limitations. First, dietary data were collected using a self-administered 24-h recall method, which may overlook important dietary details and affect the representativeness of EAT-Lancet scores. However, previous studies found a strong correlation (Pearson correlation coefficient = 0.88; *P* < 0.0001) between dietary intake reported at baseline and the average diet derived from repeated assessments between 2009 and 2012^55^. Second, although we adjusted for a wide range of potential confounders, the possibility of residual confounders cannot be ruled out due to the observational nature of the study. Third, this study has high a proportion of missing data for two key covariates: total energy intake and PA. While we addressed this issue using MICE, the validity of this approach relies on the assumption that data are missing at random (MAR), which cannot be empirically verified. The high level of missingness increases the potential for bias and reduces the precision of the estimated effects associated with these variables. Although the analysis showed that excluding these covariates did not materially alter the main findings, the possibility of residual confounding cannot be fully ruled out. Therefore, the results related to the EAT-Lancet diet and frailty risk should be interpreted with caution, particularly those involving adjustment for energy intake and PA. Finally, as participants were recruited on a voluntary basis, there is a potential for selection bias. Moreover, over 97% of the participants in our study were white, which may limit the generalizability of our findings.

In conclusion, this prospective study revealed that a higher adherence to the EAT-Lancet diet is associated with a lower risk of frailty, with metabolites playing a partial mediating role. The findings highlight the far-reaching benefits of promoting this achievable and sustainable dietary pattern for the prevention of frailty.

## Methods

### Study population

The UK Biobank study design has been thoroughly documented in previous publications^56^. In summary, the UK Biobank is a large-scale, population-based prospective cohort study aimed at examining the influence of genetic and lifestyle factors on disease. Between 2006 and 2010, the study enrolled over 500,000 individuals aged 40-69 years from 22 locations across England, Scotland, and Wales. Upon enrollment, participants completed a touchscreen questionnaire, participated in a brief verbal interview, underwent physical assessments, and provided biological samples. The study received approval from the Northwest Multi-center Research Ethics Committee (MREC) (approval number 21/NW/0157), and all participants gave informed written consent during enrollment.

The procedure for participants’ selection is shown in Supplementary Fig. 1. In brief, a total of 72,424 participants who completed at least two frailty assessments were initially included. We further excluded participants who had no information on 24-h dietary recalls (*N* = 27,835), those who withdrew from the UK Biobank (*N* = 5), and those who had been diagnosed with frailty at baseline (*N* = 119). Finally, a total of 44,465 participants were included in the current study.

### Assessment of outcomes

The health outcome of the current study was frailty, which was measured up to three times during follow-up: in 2012 to 2013, 2014 to 2022, and 2019 to 2022. Physical frailty was assessed using a modified version of the Fried phenotype^1^, which includes five key components: weight loss, exhaustion, low physical activity, slow gait speed, and low grip strength (Supplementary Table 5). As the UK Biobank used different questions and measurements compared to the original Cardiovascular Health Study on which the phenotype is based, we adjusted the criteria to align with the available data, following methods outlined in prior studies^57–59^. Frailty severity was determined by counting the number of frailty components present, resulting in a score ranging from 0 to 5. Consistent with previous research^58^, participants were classified as frail if they met three or more criteria.

### Assessment of EAT-Lancet diet score

Dietary intake was assessed using the Oxford WebQ questionnaire on up to five occasions^60,61^^.^ To justify the use of a single dietary assessment in long-term analysis, we drew on evidence within the UK Biobank cohort indicating a strong correlation (Pearson correlation coefficient = 0.88; *P* < 0.0001) between the baseline healthy Plant-based Diet Index (hPDI) and the mean hPDI derived from repeated assessments between 2009 and 2012^55^. This finding supports the relative stability of dietary pattern rankings over time. To further validate this approach, we also conducted a sensitivity analysis using the average dietary intake across all available assessments.

The EAT-Lancet diet score was calculated using the established method developed by Knuppel et al.^62^, which estimates adherence to the EAT-Lancet Commission’s recommendations for a healthy and sustainable food system. The score consisted of 8 main dietary elements (with 14 food components): whole grains, tubers and starchy vegetables, vegetables, fruits, dairy foods, protein sources, added fats, and added sugars (Supplementary Table 6). Each food component was assigned a score of 0 or 1 based on adherence to the EAT-Lancet recommendations, with cumulative scores ranging from 0 to 14, where higher scores reflect greater dietary compliance.

### Measurement of metabolic biomarkers

Metabolomic profiling was performed on nonfasting ethylenediaminetetraacetic acid (EDTA) plasma samples from approximately 120,000 UK Biobank participants using a high-throughput nuclear magnetic resonance (NMR) based metabolic biomarker profiling platform. Plasma aliquots were stored at −80 °C, thawed at 4 °C, centrifuged (3400 × *g*, 3 min), and mixed with phosphate buffer (1:1 ratio) in 3-mm NMR tubes using automated liquid handlers. Samples were analyzed between June 2019 and April 2020 on 500 MHz proton NMR spectrometers, quantifying 251 biomarkers including lipids, lipoprotein subclasses, fatty acids, amino acids, and inflammation markers through proprietary software. Quality control involved internal controls and masked duplicates in each 96-well plate, with coefficients of variation below 5% for most biomarkers. Real-time monitoring and batch consistency assessments confirmed minimal technical variability across five sample batches. Detailed methodologies are documented in the UK Biobank NMR companion file (https://biobank.ndph.ox.ac.uk/showcase/refer.cgi?id=130).

### Assessment of covariates

The covariates in this study included demographic variables such as sex (male/female), age, education level (college or university/others), and employment status (employed/unemployed), along with other potential confounders that may be associated with frailty, including energy intake (kcal), physical activity (PA, low/moderate/high), alcohol drinking (never/previous/current), smoking status (never/previous/current), Townsend Deprivation Index, and self-reported physician diagnosed: CVD (yes/no) and cancer (yes/no).

### Statistical analysis

The baseline characteristics were described as means with standard deviation (SD) for continuous variables and percentages for categorical variables. Missing values were imputed using multiple imputation by chained equations (MICE) (Supplementary Table 7).

### Association of EAT-Lancet diet with the risk of frailty

The Cox proportional hazards models were used to estimate the hazard ratio (HR) and 95% confidence interval (CI) for the associations between adherence to the EAT-Lancet diet and the risk of frailty, including its five components: exhaustion, low physical activity, slow gait speed, low grip strength, and weight loss. Additionally, we conducted a component-level analysis to examine associations between each dietary component in the EAT-Lancet score and incident frailty risk, with each component analyzed separately using the same Cox proportional hazards models as in the primary analysis. Three adjustment models were applied: Model 1 (crude); Model 2 (adjusted for age, sex, and energy intake); and Model 3 (further adjusted for PA, Townsend Deprivation Index, education level, employment status, smoking, alcohol drinking, CVD, and cancer). The proportional hazards assumption was evaluated using Schoenfeld residuals, and no violations of the assumption were found. Additionally, we used restricted cubic spline and cumulative risk curves to analyze the relationship between EAT-Lancet diet scores and the risk of frailty.

### Subgroup analysis and sensitivity analysis

Subgroup analyses were conducted to assess potential interaction effects among subgroups of sex (male, female), age (<65 years, ≥65 years), smoking status (yes, no), drinking status (yes, no), PA (low, moderate-high), and BMI (<25, ≥25), respectively. In addition, we conducted six sensitivity analyses to verify the robustness of these associations: (1) we repeated the main analysis by excluding participants diagnosed with frailty within 2 years after baseline to control reverse causation; (2) we restricted the analysis among participants without CVD and cancer at baseline; (3) we included body mass index (BMI) of participants for analysis; (4) we calculated the EAT-Lancet diet score by averaging dietary intake from all available assessments; (5) further adjusted for baseline depression status; (6) further adjusted for baseline polypharmacy.

### Identifying the metabolites reflecting adherence to EAT-Lancet diet and frailty

To identify metabolites reflecting adherence to the EAT-Lancet diet and frailty, we implemented the following preprocessing procedure: First, participants with completely missing metabolite data were excluded. Next, metabolite variables with >25% missing data were removed. For metabolites exhibiting significant skewness (absolute skewness >2), natural log transformation was applied to improve distributional normality^63^. All metabolites were then standardized to z-scores. Finally, remaining missing values were imputed using a random forest model, which leverages inter-variable correlations to generate robust estimates appropriate for the complexity of metabolomic data^64^. To account for multiple testing, *P*-values from the regression analyses were adjusted using the Benjamini-Hochberg false discovery rate (FDR) method, considering the number of metabolites tested (*n* = 251). For additional information on metabolites, see Supplementary Table 8.

To characterize the metabolic signature associated with the EAT-Lancet diet, we used elastic net penalized Cox regression with a training-testing approach. First, we performed regression analysis between EAT-Lancet diet scores and 251 metabolites. The optimal lambda parameter was selected through 10-fold cross-validation, choosing the value where the mean squared error fell with one standard error of the minimum observed value. Subsequently, we calculated a composite metabolic signature as the weighted sum of selected metabolites (those with non-zero coefficients), where each weight corresponded to the coefficient derived from the elastic net regression. This aggregate metabolic signature was identified through our analytical framework.

Finally, we evaluated the association between the metabolic signatures and frailty using Cox proportional hazards models to estimate hazard ratios. To quantify the mediating effects, we implemented a mediation analysis framework that systematically estimated the total effect of the EAT-Lancet diet on frailty, the effect of the exposure on metabolic signatures, and the effect of the mediators on frailty after adjusting for the exposure.

All analyses were performed using R (version 4.0.0, R Foundation for Statistical Computing). A two-sided *P* < 0.05 in each test was considered a statistically significant difference.

## Supplementary information

Supplementary Information

## Data Availability

All data relevant to the study were obtained using the UK Biobank resource under application number [93398]. No additional data are available.

## Code availability

The code that supports the findings of this study is available from the corresponding author upon reasonable request.

## References

[1] Fried, L. P. et al. Frailty in older adults: evidence for a phenotype. *J. Gerontol. A Biol. Sci. Med Sci.* **56**, M146–M156 (2001). 11253156 10.1093/gerona/56.3.m146

[2] Clegg, A., Young, J., Iliffe, S., Rikkert, M. O. & Rockwood, K. Frailty in elderly people. *Lancet* **381**, 752–762 (2013). 23395245 10.1016/S0140-6736(12)62167-9 PMC4098658

[3] Fhon, J. R., Rodrigues, R. A., Neira, W. F., Huayta, V. M. & Robazzi, M. L. Fall and its association with the frailty syndrome in the elderly: systematic review with meta-analysis. *Rev. Esc. Enferm. USP* **50**, 1005–1013 (2016). 28198967 10.1590/S0080-623420160000700018

[4] Li, G. et al. Frailty and risk of fractures in patients with type 2 diabetes. *Diabetes Care* **42**, 507–513 (2019). 30692240 10.2337/dc18-1965

[5] Liu, H. X. et al. Association between frailty and incident risk of disability in community-dwelling elder people: evidence from a meta-analysis. *Public Health* **175**, 90–100 (2019). 31454631 10.1016/j.puhe.2019.06.010

[6] Shi, S. M., Olivieri-Mui, B., McCarthy, E. P. & Kim, D. H. Changes in a frailty index and association with mortality. *J. Am. Geriatr. Soc.* **69**, 1057–1062 (2021). 33377190 10.1111/jgs.17002 PMC8071066

[7] Kojima, G., Iliffe, S., Jivraj, S. & Walters, K. Association between frailty and quality of life among community-dwelling older people: a systematic review and meta-analysis. *J. Epidemiol. Community Health* **70**, 716–721 (2016). 26783304 10.1136/jech-2015-206717

[8] Hoogendijk, E. O., Suanet, B., Dent, E., Deeg, D. J. & Aartsen, M. J. Adverse effects of frailty on social functioning in older adults: results from the Longitudinal Aging Study Amsterdam. *Maturitas* **83**, 45–50 (2016). 26428078 10.1016/j.maturitas.2015.09.002

[9] O’Caoimh, R. et al. Prevalence of frailty in 62 countries across the world: a systematic review and meta-analysis of population-level studies. *Age Ageing* **50**, 96–104 (2021). 33068107 10.1093/ageing/afaa219

[10] Ilinca, S. & Calciolari, S. The patterns of health care utilization by elderly Europeans: frailty and its implications for health systems. *Health Serv. Res.* **50**, 305–320 (2015). 25139146 10.1111/1475-6773.12211 PMC4319884

[11] Hoogendijk, E. O. et al. Frailty: implications for clinical practice and public health. *Lancet* **394**, 1365–1375 (2019). 31609228 10.1016/S0140-6736(19)31786-6

[12] Ghosh, T. S. et al. Mediterranean diet intervention alters the gut microbiome in older people reducing frailty and improving health status: the NU-AGE 1-year dietary intervention across five European countries. *Gut* **69**, 1218–1228 (2020). 32066625 10.1136/gutjnl-2019-319654 PMC7306987

[13] Ni Lochlainn, M. et al. Nutrition and frailty: opportunities for prevention and treatment. *Nutrients* **13**, 2349 (2021). 34371858 10.3390/nu13072349 PMC8308545

[14] Qi, R., Yang, Y., Sheng, B., Li, H. & Zhang, X. Plant-based diet indices and their association with frailty in older adults: a CLHLS-based cohort study. *Nutrients* **15**, 5120 (2023). 38140379 10.3390/nu15245120 PMC10745508

[15] Willett, W. et al. Food in the Anthropocene: the EAT-Lancet Commission on healthy diets from sustainable food systems. *Lancet* **393**, 447–492 (2019). 30660336 10.1016/S0140-6736(18)31788-4

[16] Dalile, B. et al. The EAT-Lancet reference diet and cognitive function across the life course. *Lancet Planet Health* **6**, e749–e759 (2022). 36087605 10.1016/S2542-5196(22)00123-1

[17] Berthy, F. et al. Association between adherence to the EAT-Lancet diet and risk of cancer and cardiovascular outcomes in the prospective NutriNet-Santé cohort. *Am. J. Clin. Nutr.* **116**, 980–991 (2022). 35918246 10.1093/ajcn/nqac208

[18] Zhang, S. et al. Adherence to the EAT-Lancet diet, genetic susceptibility, and risk of type 2 diabetes in Swedish adults. *Metabolism* **141**, 155401 (2023). 36682448 10.1016/j.metabol.2023.155401

[19] Zhang, S. et al. Associations of the EAT-Lancet reference diet with metabolic dysfunction-associated steatotic liver disease and its severity: a multicohort study. *Hepatology* **81**, 1583–1594 (2024). 39094016 10.1097/HEP.0000000000001039

[20] Stubbendorff, A. et al. Development of an EAT-Lancet index and its relation to mortality in a Swedish population. *Am. J. Clin. Nutr.* **115**, 705–716 (2022). 34791011 10.1093/ajcn/nqab369 PMC8895215

[21] Jacob, M., Lopata, A. L., Dasouki, M. & Abdel Rahman, A. M. Metabolomics toward personalized medicine. *Mass Spectrom. Rev.* **38**, 221–238 (2019). 29073341 10.1002/mas.21548

[22] Fernandez, C. et al. Plasma lipidome and prediction of type 2 diabetes in the population-based malmö diet and Cancer Cohort. *Diabetes Care* **43**, 366–373 (2020). 31818810 10.2337/dc19-1199

[23] Li, J. et al. The Mediterranean diet, plasma metabolome, and cardiovascular disease risk. *Eur. Heart J.* **41**, 2645–2656 (2020). 32406924 10.1093/eurheartj/ehaa209 PMC7377580

[24] León-Muñoz, L. M., García-Esquinas, E., López-García, E., Banegas, J. R. & Rodríguez-Artalejo, F. Major dietary patterns and risk of frailty in older adults: a prospective cohort study. *BMC Med.* **13**, 11 (2015). 25601152 10.1186/s12916-014-0255-6 PMC4298966

[25] Lopez-Garcia, E., Hagan, K. A., Fung, T. T., Hu, F. B. & Rodríguez-Artalejo, F. Mediterranean diet and risk of frailty syndrome among women with type 2 diabetes. *Am. J. Clin. Nutr.* **107**, 763–771 (2018). 29722845 10.1093/ajcn/nqy026

[26] Struijk, E. A. et al. Diet quality and risk of frailty among older women in the Nurses’ Health Study. *Am. J. Clin. Nutr.* **111**, 877–883 (2020). 32091575 10.1093/ajcn/nqaa028 PMC7138663

[27] Jayanama, K. et al. Relationship between diet quality scores and the risk of frailty and mortality in adults across a wide age spectrum. *BMC Med.* **19**, 64 (2021). 33722232 10.1186/s12916-021-01918-5 PMC7962372

[28] Yoh, K., Ikeda, K., Horie, K. & Inoue, S. Roles of estrogen, estrogen receptors, and estrogen-related receptors in skeletal muscle: regulation of mitochondrial function. *Int. J. Mol. Sci* **24**, 1853 (2023). 36768177 10.3390/ijms24031853 PMC9916347

[29] Cipolletti, M., Solar Fernandez, V., Montalesi, E., Marino, M. & Fiocchetti, M. Beyond the antioxidant activity of dietary polyphenols in cancer: the modulation of estrogen receptors (ERs) signaling. *Int. J. Mol. Sci* **19**, 2624 (2018). 30189583 10.3390/ijms19092624 PMC6165334

[30] Cai, H. et al. Health outcomes, environmental impacts, and diet costs of adherence to the EAT-Lancet Diet in China in 1997-2015: a health and nutrition survey. *Lancet Planet Health* **8**, e1030–e1042 (2024). 39674193 10.1016/S2542-5196(24)00285-7

[31] Wu, H. et al. Dietary pattern modifies the risk of MASLD through metabolomic signature. *JHEP Rep.* **6**, 101133 (2024). 39081700 10.1016/j.jhepr.2024.101133 PMC11286987

[32] Yao, Z. et al. Dietary patterns, metabolomics and frailty in a large cohort of 120 000 participants. *Food Funct.* **15**, 3174–3185 (2024). 38441259 10.1039/d3fo03575a

[33] Azzolino, D. et al. Omega-3 polyunsatured fatty acids and physical performance across the lifespan: a narrative review. *Front Nutr.* **11**, 1414132 (2024). 38966419 10.3389/fnut.2024.1414132 PMC11223594

[34] Troesch, B. et al. Expert opinion on benefits of long-chain omega-3 fatty acids (DHA and EPA) in aging and clinical nutrition. *Nutrients* **12**, 2555 (2020). 32846900 10.3390/nu12092555 PMC7551800

[35] Calder, P. C. et al. Health relevance of the modification of low grade inflammation in ageing (inflammageing) and the role of nutrition. *Ageing Res. Rev.* **40**, 95–119 (2017). 28899766 10.1016/j.arr.2017.09.001

[36] Dupont, J., Dedeyne, L., Dalle, S., Koppo, K. & Gielen, E. The role of omega-3 in the prevention and treatment of sarcopenia. *Aging Clin. Exp. Res.* **31**, 825–836 (2019). 30784011 10.1007/s40520-019-01146-1 PMC6583677

[37] McGlory, C., Calder, P. C. & Nunes, E. A. The influence of omega-3 fatty acids on skeletal muscle protein turnover in health, disuse, and disease. *Front. Nutr.* **6**, 144 (2019). 31555658 10.3389/fnut.2019.00144 PMC6742725

[38] Heshmati, J. et al. Omega-3 fatty acids supplementation and oxidative stress parameters: A systematic review and meta-analysis of clinical trials. *Pharm. Res* **149**, 104462 (2019). 10.1016/j.phrs.2019.104462 31563611

[39] zięgielewska-Gęsiak, S. & Muc-Wierzgoń, M. Inflammation and oxidative stress in frailty and metabolic syndromes-two sides of the same coin. *Metabolites* **13**, 475 (2023). 37110134 10.3390/metabo13040475 PMC10144989

[40] Gao, L. et al. Novel n-3 fatty acid oxidation products activate Nrf2 by destabilizing the association between Keap1 and Cullin3. *J. Biol. Chem.* **282**, 2529–2537 (2007). 17127771 10.1074/jbc.M607622200

[41] Erdogan, H. et al. Effect of fish oil supplementation on plasma oxidant/antioxidant status in rats. *Prostaglandins Leukot. Ess. Fat. Acids* **71**, 149–152 (2004). 10.1016/j.plefa.2004.02.001 15253883

[42] Musazadeh, V. et al. Can omega-3 fatty acids and vitamin E co-supplementation affect obesity indices?. *Int. J. Vitam. Nutr. Res* **93**, 471–480 (2023). 35796416 10.1024/0300-9831/a000757

[43] Ticinesi, A. et al. Aging gut microbiota at the cross-road between nutrition, physical frailty, and sarcopenia: is there a gut-muscle axis?. *Nutrients* **9**, 1303 (2017). 29189738 10.3390/nu9121303 PMC5748753

[44] Kim, M. H. & Kim, H. The roles of glutamine in the intestine and its implication in intestinal diseases. *Int. J. Mol. Sci* **18**, 1051 (2017). 28498331 10.3390/ijms18051051 PMC5454963

[45] Perna, S. et al. The role of glutamine in the complex interaction between gut microbiota and health: a narrative review. *Int. J. Mol. Sci* **20**, 5232 (2019). 31652531 10.3390/ijms20205232 PMC6834172

[46] Voulgaridou, G. et al. Increasing muscle mass in elders through diet and exercise: a literature review of recent RCTs. *Foods* **12**, 1218 (2023). 36981144 10.3390/foods12061218 PMC10048759

[47] Lee, M. H. et al. Linoleic acid attenuates denervation-induced skeletal muscle atrophy in mice through regulation of reactive oxygen species-dependent signaling. *Int. J. Mol. Sci* **23**, 4778 (2022). 35563168 10.3390/ijms23094778 PMC9105847

[48] Whelan, J. & Fritsche, K. Linoleic acid. *Adv. Nutr.* **4**, 311–312 (2013). 23674797 10.3945/an.113.003772 PMC3650500

[49] Yan, Z., Xu, Y., Li, K., Zhang, W. & Liu, L. The relationship between dietary intake of ω-3 and ω-6 fatty acids and frailty risk in middle-aged and elderly individuals: a cross-sectional study from NHANES. *Front Nutr.* **11**, 1377910 (2024). 38784137 10.3389/fnut.2024.1377910 PMC11111862

[50] Yang, G. et al. Implication of G protein-coupled receptor 43 in intestinal inflammation: a mini-review. *Front. Immunol.* **9**, 1434 (2018). 29988393 10.3389/fimmu.2018.01434 PMC6023978

[51] Nogal, A., Valdes, A. M. & Menni, C. The role of short-chain fatty acids in the interplay between gut microbiota and diet in cardio-metabolic health. *Gut Microbes* **13**, 1–24 (2021). 33764858 10.1080/19490976.2021.1897212 PMC8007165

[52] den Besten, G. et al. Short-chain fatty acids protect against high-fat diet-induced obesity via a PPARγ-dependent switch from lipogenesis to fat oxidation. *Diabetes* **64**, 2398–2408 (2015). 25695945 10.2337/db14-1213

[53] Piggott, D. A. & Tuddenham, S. The gut microbiome and frailty. *Transl. Res* **221**, 23–43 (2020). 32360945 10.1016/j.trsl.2020.03.012 PMC8487348

[54] Xie, F., Yang, W., Xing, M., Zhang, H. & Ai, L. Natural polyphenols-gut microbiota interactions and effects on glycolipid metabolism via polyphenols-gut-brain axis: a state-of-the-art review. *Trends Food Sci. Technol.* **140**, 14 (2023).

[55] Heianza, Y., Zhou, T., Sun, D., Hu, F. B. & Qi, L. Healthful plant-based dietary patterns, genetic risk of obesity, and cardiovascular risk in the UK biobank study. *Clin. Nutr.* **40**, 4694–4701 (2021). 34237696 10.1016/j.clnu.2021.06.018 PMC8338907

[56] Allen, N. E. et al. Prospective study design and data analysis in UK Biobank. *Sci. Transl. Med* **16**, eadf4428 (2024). 38198570 10.1126/scitranslmed.adf4428 PMC11127744

[57] Jiang, R. et al. Associations of physical frailty with health outcomes and brain structure in 483,033 middle-aged and older adults: a population-based study from the UK Biobank. *Lancet Digit Health* **5**, e350–e359 (2023). 37061351 10.1016/S2589-7500(23)00043-2 PMC10257912

[58] Hanlon, P. et al. Frailty and pre-frailty in middle-aged and older adults and its association with multimorbidity and mortality: a prospective analysis of 493,737 UK Biobank participants. *Lancet Public Health* **3**, e323–e332 (2018). 29908859 10.1016/S2468-2667(18)30091-4 PMC6028743

[59] Zheng, Z., Lv, Y., Rong, S., Sun, T. & Chen, L. Physical frailty, genetic predisposition, and incident Parkinson disease. *JAMA Neurol.* **80**, 455–461 (2023). 36912851 10.1001/jamaneurol.2023.0183 PMC10012040

[60] Liu, B. et al. Development and evaluation of the Oxford WebQ, a low-cost, web-based method for assessment of previous 24 h dietary intakes in large-scale prospective studies. *Public Health Nutr.* **14**, 1998–2005 (2011). 21729481 10.1017/S1368980011000942

[61] Greenwood, D. C. et al. Validation of the Oxford WebQ online 24-hour dietary questionnaire using biomarkers. *Am. J. Epidemiol.* **188**, 1858–1867 (2019). 31318012 10.1093/aje/kwz165 PMC7254925

[62] Knuppel, A., Papier, K., Key, T. J. & Travis, R. C. EAT-Lancet score and major health outcomes: the EPIC-Oxford study. *Lancet* **394**, 213–214 (2019). 31235280 10.1016/S0140-6736(19)31236-X

[63] Kim, H. Y. Statistical notes for clinical researchers: assessing normal distribution (2) using skewness and kurtosis. *Restor. Dent. Endod.* **38**, 52–54 (2013). 23495371 10.5395/rde.2013.38.1.52 PMC3591587

[64] Wei, R. et al. Missing value imputation approach for mass spectrometry-based metabolomics data. *Sci. Rep.* **8**, 663 (2018). 29330539 10.1038/s41598-017-19120-0 PMC5766532

