# Supplementary material for: Metabolic signature of EAT-Lancet diet in relation to risk of frailty: a prospective cohort study
Source: NPJ Sci Food. 2025 Nov 26;9:255. doi: 10.1038/s41538-025-00619-0 (PMC12657514; doi:10.1038/s41538-025-00619-0)
Supplement: Supplementary file 1 — Supplementary Information [file 41538_2025_619_MOESM1_ESM.docx]

**Online Supplementary Materials**

**Metabolic signature of EAT-Lancet diet in relation to risk of frailty: a prospective cohort study**

**Content**

**Supplementary Table 1.** Baseline characteristics of the participants, according to the EAT-Lancet diet score.

**Supplementary Table 2**. Association of each component of EAT-Lancet diet and frailty.

**Supplementary Table 3.** Sensitivity analyses for associations between EAT-Lancet diet score and the risk of frailty.

**Supplementary Table 4.** Association of metabolites with EAT-Lancet and frailty.

**Supplementary Table 5.** The criteria for the frailty phenotype in the UK Biobank.

**Supplementary Table 6.** EAT-Lancet diet score criteria constructed for the assessment of the EAT-Lancet diet and examples of food in the UK Biobank.

**Supplementary Table 7.** Detailed information on missing covariates.

**Supplementary Table 8.** Metabolite categories based on the UK Biobank.

**Supplementary Figure 1.** Flowchart of the study.

| **Supplementary Table 1.** Baseline characteristics of the participants, according to the EAT-Lancet diet score. | | | | | |
| --- | --- | --- | --- | --- | --- |
| **Characteristics** | **Overall (*N*=44465)** | **Categories of the EAT-Lancet diet score** | | | |
|  |  | **≤9**  **(n=9662)** | **=10**  **(n=14512)** | **=11**  **(n=14755)** | **≥12**  **(n=5536)** |
| **Female** **, n (%)** | 23098 (51.9) | 4087 (42.3) | 7253 (50.0) | 8280 (56.1) | 3478 (62.8) |
| **Age, mean (SD), y** | 55.88±7.56 | 54.94±7.64 | 55.86±7.56 | 56.38±7.48 | 56.21±7.45 |
| **BMI, mean (SD)** | 26.52±4.28 | 27.33±4.42 | 26.75±4.32 | 26.20±4.13 | 25.39±3.95 |
| **Race, white, n (%)** | 43139 (97.0) | 9378 (97.1) | 14089 (97.1) | 14334 (97.1) | 5338 (96.4) |
| **Townsend Deprivation index, mean (SD)** | -1.94±2.69 | -1.80±2.79 | -1.97±2.67 | -2.06±2.62 | -1.82±2.76 |
| **Education, n (%)** |  |  |  |  |  |
| College or University | 22155 (49.8) | 4269 (44.2) | 6997 (48.2) | 7678 (52.0) | 3211 (58.0) |
| Other | 22310 (50.2) | 5393 (55.8) | 7515 (51.8) | 7077 (48.0) | 2325 (42.0) |
| **Employment, n (%)** |  |  |  |  |  |
| No | 14688 (33.0) | 2838 (29.4) | 4813 (33.2) | 5134 (34.8) | 1903 (34.4) |
| Yes | 29777 (67.0) | 6824 (70.6) | 9699 (66.8) | 9621 (65.2) | 3633 (65.6) |
| **Physical activity, n (%)** |  |  |  |  |  |
| Low | 8193 (18.4) | 2075 (21.5) | 2806 (19.3) | 2511 (17.0) | 801 (14.5) |
| Moderate | 18887 (42.5) | 4056 (42.0) | 6207 (42.8) | 6294 (42.7) | 2330 (42.1) |
| High | 17385 (39.1) | 3531 (36.5) | 5499 (37.9) | 5950 (40.3) | 2405 (43.4) |
| **Drinking, n (%)** |  |  |  |  |  |
| Current | 42405 (95.4) | 9237 (95.6) | 13829 (95.3) | 14088 (95.5) | 5251 (94.9) |
| Previous | 978 (2.2) | 195 (2.0) | 321 (2.2) | 308 (2.1) | 154 (2.8) |
| Never | 1082 (2.4) | 230 (2.4) | 362 (2.5) | 359 (2.4) | 131 (2.4) |
| **Smoke, n (%)** |  |  |  |  |  |
| Current | 2565 (5.8) | 767 (7.9) | 889 (6.1) | 687 (4.7) | 222 (4.0) |
| Previous | 14989 (33.7) | 3356 (34.7) | 4938 (34.0) | 4839 (32.8) | 1856 (33.5) |
| Never | 26911 (60.5) | 5539 (57.3) | 8685 (59.8) | 9229 (62.5) | 3458 (62.5) |
| **Cancer, n(%)** |  |  |  |  |  |
| No | 41816 (94.0) | 9145 (94.6) | 13682 (94.3) | 13821 (93.7) | 5168 (93.4) |
| Yes | 2649 (6.0) | 517 (5.4) | 830 (5.7) | 934 (6.3) | 368 (6.6) |
| **Cardiovascular Disease, n(%)** |  |  |  |  |  |
| No | 33283 (74.9) | 7003 (72.5) | 10733 (74.0) | 11161 (75.6) | 4386 (79.2) |
| Yes | 11182 (25.1) | 2659 (27.5) | 3779 (26.0) | 3594 (24.4) | 1150 (20.8) |
| Abbreviations: BMI, body mass index | | | | | |

| **Supplementary Table 2**. Association of each component of EAT-Lancet diet and frailty. | | | |
| --- | --- | --- | --- |
| **Component** | **HR (95% CI)** | **Component** | **HR (95% CI)** |
| **Whole grains** |  | **Eggs** |  |
| Model 1^a^ | 1.18 (0.30 - 4.73) | Model 1^a^ | 0.95(0.82 - 1.09) |
| Model 2^b^ | 0.91 (0.23 - 3.65) | Model 2^b^ | 0.98(0.86 - 1.13) |
| Model 3^c^ | 0.87 (0.22 - 3.49) | Model 3^c^ | 0.99(0.86 - 1.14) |
| **Potatoes** |  | **Fish** |  |
| Model 1^a^ | 0.83 (0.68 - 1.00) | Model 1^a^ | 1.11 (0.81 - 1.53) |
| Model 2^b^ | 0.82 (0.68 - 1.00) | Model 2^b^ | 1.10 (0.80 - 1.51) |
| Model 3^c^ | 0.85 (0.70 - 1.03) | Model 3^c^ | 1.09 (0.79 - 1.50) |
| **Vegetables** |  | **Dry beans, lentils, peas** |  |
| Model 1^a^ | 0.77 (0.69 - 0.87) | Model 1^a^ | 0.89 (0.66 - 1.20) |
| Model 2^b^ | 0.71 (0.63 - 0.80) | Model 2^b^ | 0.89 (0.66 - 1.21) |
| Model 3^c^ | 0.79 (0.70 - 0.89) | Model 3^c^ | 0.95 (0.70 - 1.28) |
| **Fruits** |  | **Soy foods** |  |
| Model 1^a^ | 0.68 (0.60 - 0.77) | Model 1^a^ | 1.60 (0.60 - 4.27) |
| Model 2^b^ | 0.59 (0.52 - 0.67) | Model 2^b^ | 1.49 (0.56 - 3.98) |
| Model 3^c^ | 0.68 (0.60 - 0.78) | Model 3^c^ | 1.28 (0.48 - 3.42) |
| **Dairy foods** |  | **Peanuts or tree nuts** |  |
| Model 1^a^ | 0.82 (0.68 - 0.99) | Model 1^a^ | 0.80 (0.62 - 1.03) |
| Model 2^b^ | 0.78 (0.65 - 0.94) | Model 2^b^ | 0.76 (0.59 - 0.98) |
| Model 3^c^ | 0.79 (0.65 - 0.95) | Model 3^c^ | 0.83 (0.65 - 1.07) |
| **Beef, lamb, pork** |  | **Added fats** |  |
| Model 1^a^ | 0.82 (0.73 - 0.92) | Model 1^a^ | 0.63 (0.44 - 0.90) |
| Model 2^b^ | 0.82 (0.73 - 0.92) | Model 2^b^ | 0.60 (0.42 - 0.86) |
| Model 3^c^ | 0.86 (0.76 - 0.97) | Model 3^c^ | 0.66 (0.46 - 0.94) |
| **Chicken, other poultry** |  | **Add sugar** |  |
| Model 1^a^ | 1.04 (0.90 - 1.19) | Model 1^a^ | 1.65 (1.12 - 2.43) |
| Model 2^b^ | 0.98 (0.85 - 1.13) | Model 2^b^ | 1.81 (1.23 - 2.67) |
| Model 3^c^ | 1.01 (0.87 - 1.16) | Model 3^c^ | 1.55 (1.05 - 2.29) |
| ^a^ Original model without adjusting for any variables.  ^b^ Adjusted for age, sex and energy.  ^c^ Adjusted for age, sex, and energy, PA, education, employment, smoking, drinking, Townsend deprivation index, CVD and cancer. | | | |

| **Supplementary Table 3.** Sensitivity analyses for associations between EAT-Lancet diet score and the risk of frailty. | | | | |
| --- | --- | --- | --- | --- |
|  | **Categories of the EAT-Lancet diet score** | | | |
|  | **≤9** | **=10** | **=11** | **≥12** |
| **Excluding participants diagnosed within 2 years after baseline^*1^** | | | | |
| Cases/Person-Years | 335/87897 | 375/132397 | 324/134296 | 86/50229 |
| Adjusted HR (95% CI) | Ref | 0.75 (0.64 - 0.87) | 0.65 (0.55 - 0.76) | 0.51 (0.40 - 0.64) |
| **Excluding individuals with cardiovascular disease and cancer at baseline^*2^** | | | | |
| Cases/Person-Years | 171/61723 | 199/94625 | 179/96870 | 47/37774 |
| Adjusted HR (95% CI) | Ref | 0.72 (0.59 - 0.89) | 0.64 (0.52 - 0.80) | 0.45 (0.32 - 0.62) |
| **Including individuals’ BMI at baseline^*3^** | | | | |
| Cases/Person-Years | 335/87897 | 375/132397 | 324/134296 | 86/50229 |
| Adjusted HR (95% CI) | Ref | 0.81 (0.69 - 0.94) | 0.71 (0.61 - 0.84) | 0.64 (0.51 - 0.82) |
| **Using the average dietary intake across all available assessments*^4^** | | | |  |
| Cases/Person-Years | 65/18052 | 965/344428 | 72/30522 | 18/11818 |
| Adjusted HR (95% CI) | Ref | 0.64 (0.50 - 0.83) | 0.68 (0.48 - 0.95) | 0.47 (0.28 - 0.79) |
| **Adjusted for baseline depression*^5^** | |  |  |  |
| Cases/Person-Years | 335/87897 | 375/132397 | 324/134296 | 86/50229 |
| Adjusted HR (95% CI) | 0.75 (0.65 - 0.87) | 0.65 (0.56 - 0.76) | 0.51 (0.40 - 0.65) | 0.75 (0.65 - 0.87) |
| **Adjusted for baseline polypharmacy*^6^** | |  |  |  |
| Cases/Person-Years | 335/87897 | 375/132397 | 324/134296 | 86/50229 |
| Adjusted HR (95% CI) | 0.76 (0.65 - 0.88) | 0.66 (0.57 - 0.77) | 0.52 (0.41 - 0.67) | 0.76 (0.65 - 0.88) |
| Abbreviations: Ref, reference.  ^*1^Adjusted for age, sex, energy, PA, education, employment, smoking, drinking, Townsend deprivation index, BMI, CVD and cancer.  ^*2^ Adjusted for age, sex, energy, PA, education, employment, smoking, drinking, Townsend deprivation index and BMI.  ^*3^ Adjusted for age, sex, energy, PA, education, employment, smoking, drinking, Townsend deprivation index, CVD, cancer, and BMI.  ^*4^ Adjusted for age, sex, energy, PA, education, employment, smoking, drinking, Townsend deprivation index, CVD and cancer.  ^*5^ Adjusted for age, sex, energy, PA, education, employment, smoking, drinking, Townsend deprivation index, CVD, cancer, and depression.  ^*6^ Adjusted for age, sex, energy, PA, education, employment, smoking, drinking, Townsend deprivation index, CVD, cancer, and polypharmacy. | | | | |

| **Supplementary Table 4.** Association of metabolites with EAT-Lancet and frailty. | | | | | |
| --- | --- | --- | --- | --- | --- |
|  | **Eat-Lancet diet** | | | **Frailty** | |
| **Metabolites** | **Beta** | **95% CI** | **Adjusted *P* value** | **HR (95% CI)** | **Adjusted *P* value** |
| SCA | 0.002 | (-0.0084 , 0.0124) | 0.726742911 | 1.0841(1.0107 - 1.1628) | 0.035288046 |
| Total_C | -0.0093 | (-0.0191 , 5e-04) | 0.074561497 | 0.8914(0.8265 - 0.9614) | 0.004605972 |
| non_HDL_C | -0.0174 | (-0.0275 , -0.0072) | 0.001177352 | 0.9838(0.9156 - 1.057) | 0.690791443 |
| Remnant_C | -0.0194 | (-0.0296 , -0.0093) | 0.000269965 | 1.006(0.9369 - 1.0803) | 0.879233264 |
| VLDL_C | -0.0239 | (-0.0342 , -0.0136) | 1.03E-05 | 1.1105(1.0364 - 1.19) | 0.004715548 |
| Clinical_LDL_C | -0.0135 | (-0.0235 , -0.0034) | 0.011491474 | 0.9478(0.8815 - 1.0191) | 0.182473291 |
| LDL_C | -0.0148 | (-0.0249 , -0.0046) | 0.005922478 | 0.9633(0.8961 - 1.0355) | 0.354578573 |
| HDL_C | 0.0179 | (0.0085 , 0.0272) | 0.00029274 | 0.6797(0.619 - 0.7463) | 6.51E-15 |
| Total_TG | -0.0374 | (-0.0473 , -0.0276) | 4.59E-13 | 1.2308(1.1535 - 1.3133) | 1.19E-09 |
| VLDL_TG | -0.0355 | (-0.0453 , -0.0257) | 4.73E-12 | 1.2343(1.1562 - 1.3177) | 9.52E-10 |
| LDL_TG | -0.0374 | (-0.0474 , -0.0275) | 6.42E-13 | 1.2054(1.1323 - 1.2832) | 1.29E-08 |
| HDL_TG | -0.0416 | (-0.0516 , -0.0316) | 2.84E-15 | 1.1475(1.0741 - 1.2258) | 8.91E-05 |
| Total_PL | -0.0197 | (-0.0294 , -0.01) | 0.000125521 | 0.9213(0.8533 - 0.9948) | 0.052096585 |
| VLDL_PL | -0.0308 | (-0.041 , -0.0206) | 6.83E-09 | 1.1849(1.1072 - 1.268) | 2.09E-06 |
| LDL_PL | -0.0183 | (-0.0285 , -0.0082) | 0.00064337 | 0.9867(0.9185 - 1.06) | 0.73417212 |
| HDL_PL | -0.0021 | (-0.0115 , 0.0073) | 0.682676153 | 0.7724(0.7074 - 0.8434) | 2.25E-08 |
| Total_CE | -0.0085 | (-0.0183 , 0.0012) | 0.098954045 | 0.8756(0.8113 - 0.9449) | 0.001130809 |
| VLDL_CE | -0.0202 | (-0.0305 , -0.0099) | 0.000197824 | 1.0749(1.0025 - 1.1524) | 0.059847376 |
| LDL_CE | -0.0175 | (-0.0276 , -0.0073) | 0.001191363 | 0.9842(0.916 - 1.0576) | 0.694933451 |
| HDL_CE | 0.019 | (0.0096 , 0.0284) | 0.000126342 | 0.676(0.6162 - 0.7415) | 1.88E-15 |
| Total_FC | -0.0109 | (-0.0209 , -0.001) | 0.037259256 | 0.9326(0.8661 - 1.0042) | 0.087991856 |
| VLDL_FC | -0.0279 | (-0.0381 , -0.0176) | 2.06E-07 | 1.1555(1.0791 - 1.2374) | 7.02E-05 |
| LDL_FC | -0.007 | (-0.017 , 0.003) | 0.186591852 | 0.9086(0.8442 - 0.978) | 0.016130409 |
| HDL_FC | 0.0134 | (0.004 , 0.0227) | 0.006744776 | 0.713(0.6491 - 0.7832) | 1.01E-11 |
| Total_L | -0.0241 | (-0.0341 , -0.0141) | 4.91E-06 | 0.999(0.9295 - 1.0737) | 0.981608892 |
| VLDL_L | -0.0333 | (-0.0434 , -0.0232) | 2.39E-10 | 1.2033(1.1251 - 1.2869) | 1.70E-07 |
| LDL_L | -0.0177 | (-0.0279 , -0.0075) | 0.001003832 | 0.9845(0.9162 - 1.0578) | 0.696457064 |
| HDL_L | 0.0049 | (-0.0045 , 0.0143) | 0.334476791 | 0.7364(0.6729 - 0.8059) | 1.38E-10 |
| Total_P | -0.0155 | (-0.0252 , -0.0059) | 0.002348344 | 0.8135(0.7503 - 0.8821) | 1.32E-06 |
| VLDL_P | -0.0268 | (-0.0371 , -0.0165) | 6.66E-07 | 1.1469(1.071 - 1.2282) | 0.000167919 |
| LDL_P | -0.0118 | (-0.022 , -0.0016) | 0.029542188 | 1.023(0.9534 - 1.0978) | 0.572564901 |
| HDL_P | -0.0138 | (-0.0234 , -0.0042) | 0.006788522 | 0.7993(0.7365 - 0.8675) | 2.04E-07 |
| VLDL_size | -0.0372 | (-0.0468 , -0.0275) | 2.39E-13 | 1.2848(1.1898 - 1.3875) | 6.03E-10 |
| LDL_size | 0.0197 | (0.0096 , 0.0298) | 0.000212848 | 0.8882(0.8271 - 0.9537) | 0.001901031 |
| HDL_size | 0.0434 | (0.034 , 0.0528) | 1.87E-18 | 0.6773(0.6163 - 0.7443) | 6.51E-15 |
| Phosphoglyc | -0.0208 | (-0.0305 , -0.0112) | 4.35E-05 | 0.924(0.8549 - 0.9987) | 0.064821886 |
| TG_by_PG | -0.0343 | (-0.0439 , -0.0248) | 5.74E-12 | 1.3479(1.256 - 1.4467) | 1.94E-15 |
| Cholines | -0.0142 | (-0.0238 , -0.0046) | 0.005429038 | 0.8882(0.8208 - 0.9612) | 0.0051605 |
| Phosphatidylc | -0.015 | (-0.0246 , -0.0054) | 0.003065186 | 0.883(0.8154 - 0.9561) | 0.003570424 |
| Sphingomyelins | -0.0114 | (-0.0209 , -0.0018) | 0.024990564 | 0.8794(0.8123 - 0.952) | 0.002540031 |
| ApoB | -0.015 | (-0.0252 , -0.0048) | 0.005429038 | 1.029(0.959 - 1.104) | 0.475804636 |
| ApoA1 | -0.0047 | (-0.0142 , 0.0048) | 0.362486888 | 0.765(0.7018 - 0.8339) | 3.41E-09 |
| ApoB_by_ApoA1 | -0.0102 | (-0.0203 , -1e-04) | 0.056774975 | 1.1432(1.0665 - 1.2253) | 0.000294333 |
| Total_FA | -0.0353 | (-0.0454 , -0.0252) | 1.99E-11 | 1.1261(1.0521 - 1.2052) | 0.001107171 |
| Unsaturation | 0.0553 | (0.0455 , 0.0652) | 1.62E-26 | 0.7358(0.6823 - 0.7935) | 1.52E-14 |
| Omega_3 | 0.0078 | (-0.0025 , 0.0182) | 0.155008972 | 0.9273(0.8586 - 1.0015) | 0.075537719 |
| Omega_6 | -0.0047 | (-0.0149 , 0.0055) | 0.40053852 | 0.9886(0.9189 - 1.0636) | 0.774980254 |
| PUFA | -0.0019 | (-0.0121 , 0.0083) | 0.7349029 | 0.9706(0.9009 - 1.0456) | 0.47934936 |
| MUFA | -0.0444 | (-0.0543 , -0.0346) | 8.07E-18 | 1.2333(1.1582 - 1.3132) | 2.55E-10 |
| SFA | -0.0489 | (-0.0589 , -0.0389) | 8.93E-21 | 1.1301(1.0573 - 1.2079) | 0.00058725 |
| LA | 0.0124 | (0.0021 , 0.0226) | 0.022600973 | 0.981(0.9118 - 1.0553) | 0.647600721 |
| DHA | 0.0289 | (0.0187 , 0.0391) | 6.77E-08 | 0.8154(0.7506 - 0.8859) | 3.04E-06 |
| Omega_3_pct | 0.032 | (0.0215 , 0.0424) | 5.55E-09 | 0.855(0.7899 - 0.9256) | 0.000205143 |
| Omega_6_pct | 0.0664 | (0.0567 , 0.0761) | 5.61E-39 | 0.7728(0.7221 - 0.827) | 6.93E-13 |
| PUFA_pct | 0.077 | (0.0673 , 0.0867) | 2.44E-52 | 0.7384(0.6905 - 0.7896) | 2.14E-17 |
| MUFA_pct | -0.0521 | (-0.0617 , -0.0425) | 3.58E-25 | 1.4492(1.3528 - 1.5525) | 1.12E-23 |
| SFA_pct | -0.0773 | (-0.0876 , -0.0669) | 2.08E-46 | 1.0994(1.0257 - 1.1783) | 0.011553178 |
| LA_pct | 0.0799 | (0.0702 , 0.0896) | 7.67E-56 | 0.7707(0.7166 - 0.8288) | 1.25E-11 |
| DHA_pct | 0.0536 | (0.0433 , 0.064) | 6.20E-23 | 0.7647(0.7053 - 0.8292) | 3.34E-10 |
| PUFA_by_MUFA | 0.0673 | (0.0575 , 0.0772) | 4.10E-39 | 0.6776(0.6278 - 0.7314) | 1.39E-21 |
| Ala | -6.00E-04 | (-0.0112 , 0.0099) | 0.918923651 | 1.0666(0.9948 - 1.1435) | 0.093461165 |
| Gln | 0.0373 | (0.0269 , 0.0477) | 7.49E-12 | 0.8682(0.8073 - 0.9337) | 0.000259544 |
| Gly | 0.0382 | (0.0281 , 0.0483) | 5.60E-13 | 0.941(0.8708 - 1.0168) | 0.159430952 |
| Total_BCAA | -0.051 | (-0.0612 , -0.0408) | 1.45E-21 | 1.1122(1.039 - 1.1905) | 0.003591132 |
| Ile | -0.0418 | (-0.0522 , -0.0314) | 2.05E-14 | 1.0885(1.0177 - 1.1642) | 0.020188916 |
| Leu | -0.0481 | (-0.0583 , -0.0378) | 2.94E-19 | 1.0707(0.9986 - 1.1479) | 0.075537719 |
| Val | -0.0529 | (-0.0631 , -0.0428) | 3.15E-23 | 1.1428(1.0675 - 1.2234) | 0.000233922 |
| Tyr | -0.0324 | (-0.0429 , -0.0219) | 4.11E-09 | 1.1481(1.0735 - 1.2279) | 0.000110003 |
| Lactate | -0.0175 | (-0.0279 , -0.0071) | 0.001480839 | 1.0697(1.0017 - 1.1423) | 0.062697036 |
| Citrate | 0.0381 | (0.0278 , 0.0485) | 2.05E-12 | 1.0381(0.9652 - 1.1166) | 0.356953914 |
| Albumin | 0.0286 | (0.0183 , 0.039) | 1.33E-07 | 0.7772(0.7229 - 0.8355) | 4.41E-11 |
| GlycA | -0.0475 | (-0.0574 , -0.0375) | 1.30E-19 | 1.392(1.3074 - 1.482) | 5.67E-23 |
| XXL_VLDL_P | -0.0355 | (-0.0451 , -0.0259) | 1.74E-12 | 1.2302(1.1557 - 1.3094) | 3.15E-10 |
| XXL_VLDL_L | -0.0351 | (-0.0447 , -0.0255) | 2.94E-12 | 1.2235(1.1497 - 1.302) | 7.43E-10 |
| XXL_VLDL_PL | -0.035 | (-0.0446 , -0.0254) | 3.15E-12 | 1.2321(1.1572 - 1.3119) | 2.87E-10 |
| XXL_VLDL_C | -0.0364 | (-0.0461 , -0.0266) | 1.03E-12 | 1.232(1.1548 - 1.3142) | 8.99E-10 |
| XXL_VLDL_CE | -0.0376 | (-0.0474 , -0.0278) | 2.63E-13 | 1.2338(1.1557 - 1.3172) | 1.05E-09 |
| XXL_VLDL_FC | -0.0343 | (-0.044 , -0.0246) | 1.14E-11 | 1.2259(1.1502 - 1.3065) | 1.22E-09 |
| XXL_VLDL_TG | -0.0342 | (-0.0437 , -0.0246) | 7.61E-12 | 1.2148(1.1428 - 1.2914) | 1.43E-09 |
| XL_VLDL_P | -0.0377 | (-0.0475 , -0.0279) | 2.36E-13 | 1.2487(1.1688 - 1.3341) | 2.05E-10 |
| XL_VLDL_L | -0.0375 | (-0.0473 , -0.0277) | 3.08E-13 | 1.245(1.1656 - 1.3299) | 2.97E-10 |
| XL_VLDL_PL | -0.0358 | (-0.0456 , -0.0259) | 3.93E-12 | 1.242(1.1622 - 1.3274) | 6.03E-10 |
| XL_VLDL_C | -0.0337 | (-0.0438 , -0.0236) | 1.54E-10 | 1.2174(1.1368 - 1.3038) | 4.71E-08 |
| XL_VLDL_CE | -0.0323 | (-0.0425 , -0.0221) | 1.37E-09 | 1.1931(1.1132 - 1.2789) | 1.39E-06 |
| XL_VLDL_FC | -0.034 | (-0.0439 , -0.0241) | 5.37E-11 | 1.2327(1.1527 - 1.3182) | 2.93E-09 |
| XL_VLDL_TG | -0.0385 | (-0.0482 , -0.0288) | 4.64E-14 | 1.247(1.1689 - 1.3302) | 1.03E-10 |
| L_VLDL_P | -0.0346 | (-0.0445 , -0.0247) | 2.64E-11 | 1.2352(1.1546 - 1.3214) | 2.57E-09 |
| L_VLDL_L | -0.0342 | (-0.0442 , -0.0242) | 5.37E-11 | 1.2295(1.149 - 1.3156) | 6.29E-09 |
| L_VLDL_PL | -0.0361 | (-0.046 , -0.0262) | 3.70E-12 | 1.2483(1.1663 - 1.3362) | 6.03E-10 |
| L_VLDL_C | -0.0309 | (-0.0411 , -0.0208) | 5.87E-09 | 1.2097(1.1287 - 1.2965) | 1.82E-07 |
| L_VLDL_CE | -0.0256 | (-0.0359 , -0.0153) | 2.23E-06 | 1.1717(1.0928 - 1.2564) | 1.76E-05 |
| L_VLDL_FC | -0.0351 | (-0.0451 , -0.0251) | 1.71E-11 | 1.2396(1.1575 - 1.3274) | 2.49E-09 |
| L_VLDL_TG | -0.034 | (-0.0439 , -0.0241) | 5.37E-11 | 1.2224(1.1438 - 1.3064) | 8.49E-09 |
| M_VLDL_P | -0.0201 | (-0.0305 , -0.0098) | 0.000213496 | 1.0999(1.0261 - 1.179) | 0.011308861 |
| M_VLDL_L | -0.0247 | (-0.035 , -0.0144) | 4.89E-06 | 1.131(1.0556 - 1.2119) | 0.000865567 |
| M_VLDL_PL | -0.0201 | (-0.0304 , -0.0098) | 0.000217905 | 1.0913(1.0181 - 1.1698) | 0.020335144 |
| M_VLDL_C | -0.0046 | (-0.0147 , 0.0055) | 0.403140688 | 0.9863(0.9183 - 1.0593) | 0.727850523 |
| M_VLDL_CE | 0.0035 | (-0.0064 , 0.0135) | 0.512688909 | 0.9325(0.867 - 1.0029) | 0.082308469 |
| M_VLDL_FC | -0.0148 | (-0.0251 , -0.0045) | 0.006623604 | 1.0569(0.9854 - 1.1335) | 0.157157569 |
| M_VLDL_TG | -0.0326 | (-0.0427 , -0.0225) | 5.56E-10 | 1.2071(1.128 - 1.2918) | 1.35E-07 |
| S_VLDL_P | -0.0257 | (-0.0359 , -0.0154) | 1.85E-06 | 1.1787(1.1007 - 1.2623) | 5.34E-06 |
| S_VLDL_L | -0.0271 | (-0.0374 , -0.0169) | 4.80E-07 | 1.1712(1.0934 - 1.2544) | 1.37E-05 |
| S_VLDL_PL | -0.0211 | (-0.0314 , -0.0107) | 0.000112199 | 1.1119(1.0374 - 1.1918) | 0.004448617 |
| S_VLDL_C | -0.0202 | (-0.0305 , -0.0099) | 0.000212848 | 1.0955(1.0219 - 1.1744) | 0.015688578 |
| S_VLDL_CE | -0.0228 | (-0.0331 , -0.0125) | 2.81E-05 | 1.1173(1.0424 - 1.1976) | 0.002877436 |
| S_VLDL_FC | -0.0152 | (-0.0255 , -0.0049) | 0.005429038 | 1.0564(0.9849 - 1.1331) | 0.159927467 |
| S_VLDL_TG | -0.0307 | (-0.0407 , -0.0207) | 4.15E-09 | 1.2321(1.1523 - 1.3173) | 2.93E-09 |
| XS_VLDL_P | -0.0203 | (-0.0304 , -0.0101) | 0.000150809 | 1.0636(0.9913 - 1.1411) | 0.112626994 |
| XS_VLDL_L | -0.0201 | (-0.0303 , -0.01) | 0.000165323 | 1.0656(0.993 - 1.1436) | 0.102751764 |
| XS_VLDL_PL | -0.0195 | (-0.0297 , -0.0093) | 0.000272157 | 1.0982(1.0245 - 1.1772) | 0.012684426 |
| XS_VLDL_C | -0.0113 | (-0.0212 , -0.0014) | 0.031739036 | 0.9685(0.8997 - 1.0425) | 0.441193121 |
| XS_VLDL_CE | -0.009 | (-0.0188 , 8e-04) | 0.08294417 | 0.9359(0.8684 - 1.0086) | 0.108531887 |
| XS_VLDL_FC | -0.0161 | (-0.0262 , -0.006) | 0.002625474 | 1.0451(0.9734 - 1.1221) | 0.262569693 |
| XS_VLDL_TG | -0.0328 | (-0.0428 , -0.0228) | 2.89E-10 | 1.2149(1.1395 - 1.2953) | 7.25E-09 |
| IDL_P | -0.0174 | (-0.0274 , -0.0074) | 0.000980688 | 0.9785(0.9106 - 1.0514) | 0.593265209 |
| IDL_L | -0.0124 | (-0.0222 , -0.0027) | 0.016130753 | 0.911(0.8449 - 0.9823) | 0.022551139 |
| IDL_PL | -0.007 | (-0.0168 , 0.0027) | 0.176644027 | 0.9181(0.8515 - 0.9899) | 0.038071534 |
| IDL_C | -0.0103 | (-0.02 , -6e-04) | 0.045366989 | 0.881(0.8166 - 0.9504) | 0.001847876 |
| IDL_CE | -0.0123 | (-0.022 , -0.0027) | 0.016130753 | 0.8822(0.8178 - 0.9518) | 0.002079967 |
| IDL_FC | -0.0041 | (-0.0138 , 0.0056) | 0.433928693 | 0.8806(0.8164 - 0.9498) | 0.001747373 |
| IDL_TG | -0.035 | (-0.045 , -0.025) | 1.99E-11 | 1.1911(1.117 - 1.2701) | 2.25E-07 |
| L_LDL_P | -0.0076 | (-0.0178 , 0.0025) | 0.156185698 | 1.0113(0.9421 - 1.0855) | 0.774980254 |
| L_LDL_L | -0.0143 | (-0.0243 , -0.0042) | 0.007514479 | 0.9486(0.8817 - 1.0205) | 0.190295953 |
| L_LDL_PL | -0.0165 | (-0.0266 , -0.0064) | 0.001923345 | 0.9472(0.8808 - 1.0187) | 0.179158954 |
| L_LDL_C | -0.0107 | (-0.0208 , -6e-04) | 0.044670699 | 0.9286(0.8627 - 0.9996) | 0.067907506 |
| L_LDL_CE | -0.0125 | (-0.0226 , -0.0023) | 0.019977306 | 0.945(0.8783 - 1.0168) | 0.165206045 |
| L_LDL_FC | -0.0056 | (-0.0155 , 0.0043) | 0.290233392 | 0.8859(0.8221 - 0.9546) | 0.002498798 |
| L_LDL_TG | -0.0382 | (-0.0481 , -0.0282) | 3.16E-13 | 1.1963(1.123 - 1.2745) | 7.25E-08 |
| M_LDL_P | -0.0188 | (-0.0291 , -0.0085) | 0.000536409 | 1.0332(0.9628 - 1.1089) | 0.410291353 |
| M_LDL_L | -0.0247 | (-0.035 , -0.0145) | 4.57E-06 | 1.0413(0.9702 - 1.1176) | 0.304100909 |
| M_LDL_PL | -0.0246 | (-0.0349 , -0.0143) | 5.28E-06 | 1.0412(0.97 - 1.1176) | 0.305121036 |
| M_LDL_C | -0.0228 | (-0.0331 , -0.0126) | 2.36E-05 | 1.024(0.9538 - 1.0993) | 0.55951796 |
| M_LDL_CE | -0.0263 | (-0.0366 , -0.016) | 1.07E-06 | 1.0514(0.9798 - 1.1282) | 0.197473405 |
| M_LDL_FC | -0.0117 | (-0.0219 , -0.0015) | 0.029936024 | 0.9488(0.8826 - 1.02) | 0.188478824 |
| M_LDL_TG | -0.0359 | (-0.0457 , -0.026) | 3.70E-12 | 1.2115(1.1387 - 1.2889) | 3.70E-09 |
| S_LDL_P | -0.0147 | (-0.0249 , -0.0044) | 0.006701163 | 1.0539(0.9827 - 1.1302) | 0.177277786 |
| S_LDL_L | -0.0172 | (-0.0274 , -0.007) | 0.001406533 | 1.0479(0.9771 - 1.1238) | 0.226114004 |
| S_LDL_PL | -0.0095 | (-0.0196 , 7e-04) | 0.078204444 | 1.0388(0.9687 - 1.114) | 0.329091281 |
| S_LDL_C | -0.0171 | (-0.0273 , -0.0069) | 0.001503471 | 1.0255(0.9556 - 1.1004) | 0.531310004 |
| S_LDL_CE | -0.0219 | (-0.0321 , -0.0117) | 4.88E-05 | 1.0544(0.9831 - 1.1309) | 0.173857483 |
| S_LDL_FC | -0.0027 | (-0.0127 , 0.0074) | 0.635220088 | 0.948(0.8826 - 1.0182) | 0.178699762 |
| S_LDL_TG | -0.0332 | (-0.043 , -0.0234) | 8.02E-11 | 1.2188(1.1454 - 1.2969) | 1.41E-09 |
| XL_HDL_P | 0.0447 | (0.0351 , 0.0542) | 6.87E-19 | 0.7072(0.6413 - 0.7799) | 2.18E-11 |
| XL_HDL_L | 0.0469 | (0.0373 , 0.0565) | 1.16E-20 | 0.7024(0.6367 - 0.775) | 1.10E-11 |
| XL_HDL_PL | 0.0465 | (0.0369 , 0.0561) | 3.53E-20 | 0.7075(0.6414 - 0.7804) | 2.53E-11 |
| XL_HDL_C | 0.0486 | (0.0391 , 0.0582) | 2.52E-22 | 0.6908(0.6266 - 0.7616) | 7.39E-13 |
| XL_HDL_CE | 0.0489 | (0.0394 , 0.0584) | 1.20E-22 | 0.6754(0.6117 - 0.7458) | 7.10E-14 |
| XL_HDL_FC | 0.0457 | (0.036 , 0.0554) | 3.35E-19 | 0.7655(0.7002 - 0.8368) | 1.10E-08 |
| XL_HDL_TG | -0.0115 | (-0.0216 , -0.0015) | 0.030584375 | 1.0784(1.0074 - 1.1543) | 0.043023016 |
| L_HDL_P | 0.0372 | (0.0278 , 0.0466) | 4.64E-14 | 0.6474(0.5849 - 0.7165) | 8.16E-16 |
| L_HDL_L | 0.0345 | (0.0251 , 0.0439) | 2.62E-12 | 0.6607(0.5977 - 0.7304) | 6.51E-15 |
| L_HDL_PL | 0.029 | (0.0196 , 0.0384) | 3.44E-09 | 0.6814(0.6178 - 0.7515) | 1.28E-13 |
| L_HDL_C | 0.041 | (0.0316 , 0.0505) | 9.62E-17 | 0.6424(0.5806 - 0.7109) | 2.19E-16 |
| L_HDL_CE | 0.0424 | (0.0329 , 0.0518) | 1.04E-17 | 0.638(0.5767 - 0.7057) | 5.99E-17 |
| L_HDL_FC | 0.0361 | (0.0267 , 0.0455) | 2.61E-13 | 0.6658(0.6021 - 0.7364) | 2.21E-14 |
| L_HDL_TG | -0.0136 | (-0.0236 , -0.0036) | 0.010158678 | 0.9772(0.9067 - 1.0532) | 0.58778566 |
| M_HDL_P | -0.0076 | (-0.0171 , 0.0019) | 0.130467099 | 0.7748(0.7116 - 0.8435) | 1.09E-08 |
| M_HDL_L | -0.0139 | (-0.0234 , -0.0043) | 0.005922478 | 0.8084(0.7438 - 0.8786) | 1.30E-06 |
| M_HDL_PL | -0.0201 | (-0.0296 , -0.0106) | 6.30E-05 | 0.8466(0.7803 - 0.9185) | 0.000121681 |
| M_HDL_C | -0.0016 | (-0.0111 , 0.0079) | 0.767562115 | 0.7485(0.6869 - 0.8156) | 1.73E-10 |
| M_HDL_CE | -0.0011 | (-0.0106 , 0.0084) | 0.839456848 | 0.7473(0.6861 - 0.8138) | 1.03E-10 |
| M_HDL_FC | -0.0035 | (-0.0129 , 0.006) | 0.500820935 | 0.7627(0.699 - 0.8321) | 3.30E-09 |
| M_HDL_TG | -0.0473 | (-0.0573 , -0.0372) | 2.65E-19 | 1.1457(1.0717 - 1.2247) | 0.000124381 |
| S_HDL_P | -0.0448 | (-0.0551 , -0.0346) | 7.91E-17 | 0.9769(0.9079 - 1.0511) | 0.574125716 |
| S_HDL_L | -0.0531 | (-0.0632 , -0.043) | 1.67E-23 | 1.0171(0.9461 - 1.0934) | 0.684298758 |
| S_HDL_PL | -0.0541 | (-0.0641 , -0.0441) | 7.49E-25 | 1.0164(0.9455 - 1.0926) | 0.691827519 |
| S_HDL_C | -0.0404 | (-0.0506 , -0.0301) | 6.39E-14 | 0.9471(0.8798 - 1.0197) | 0.183448764 |
| S_HDL_CE | -0.0393 | (-0.0496 , -0.029) | 3.42E-13 | 0.9442(0.8772 - 1.0163) | 0.161068993 |
| S_HDL_FC | -0.0375 | (-0.0476 , -0.0275) | 8.87E-13 | 0.9636(0.8946 - 1.038) | 0.371353482 |
| S_HDL_TG | -0.0499 | (-0.0597 , -0.0401) | 3.10E-22 | 1.2662(1.1841 - 1.354) | 2.72E-11 |
| XXL_VLDL_PL_pct | -0.0181 | (-0.0305 , -0.0057) | 0.005840025 | 1.0719(0.9926 - 1.1577) | 0.101908709 |
| XXL_VLDL_TG_pct | -0.001 | (-0.013 , 0.0111) | 0.888738869 | 1.0469(0.9693 - 1.1306) | 0.284412303 |
| XL_VLDL_C_pct | 0.0382 | (0.0273 , 0.0491) | 1.99E-11 | 0.7789(0.7087 - 0.8562) | 5.24E-07 |
| XL_VLDL_FC_pct | 0.0405 | (0.0293 , 0.0516) | 3.90E-12 | 0.8148(0.7511 - 0.8838) | 1.77E-06 |
| XL_VLDL_TG_pct | -0.0322 | (-0.0431 , -0.0213) | 1.71E-08 | 1.2056(1.1112 - 1.3079) | 1.45E-05 |
| L_VLDL_C_pct | 0.0231 | (0.0128 , 0.0335) | 2.26E-05 | 0.8725(0.8094 - 0.9405) | 0.000676435 |
| L_VLDL_CE_pct | 0.0359 | (0.0258 , 0.0459) | 7.28E-12 | 0.8071(0.7461 - 0.8732) | 2.25E-07 |
| L_VLDL_FC_pct | -0.0162 | (-0.027 , -0.0054) | 0.00464184 | 1.0728(0.9925 - 1.1597) | 0.101908709 |
| L_VLDL_TG_pct | 0.0049 | (-0.0059 , 0.0158) | 0.402972602 | 0.9994(0.9272 - 1.0771) | 0.9865847 |
| M_VLDL_PL_pct | 0.0194 | (0.0099 , 0.029) | 0.000109764 | 0.8417(0.7854 - 0.9022) | 2.45E-06 |
| M_VLDL_C_pct | 0.0369 | (0.0275 , 0.0463) | 8.10E-14 | 0.7726(0.7181 - 0.8312) | 2.50E-11 |
| M_VLDL_CE_pct | 0.0388 | (0.0293 , 0.0482) | 4.51E-15 | 0.7642(0.7098 - 0.8227) | 5.55E-12 |
| M_VLDL_FC_pct | 0.0301 | (0.0206 , 0.0395) | 1.21E-09 | 0.8048(0.7495 - 0.8642) | 6.39E-09 |
| M_VLDL_TG_pct | -0.0337 | (-0.0431 , -0.0243) | 7.24E-12 | 1.2762(1.1868 - 1.3723) | 2.02E-10 |
| S_VLDL_PL_pct | 0.0276 | (0.0179 , 0.0373) | 5.08E-08 | 0.7966(0.739 - 0.8586) | 7.73E-09 |
| S_VLDL_C_pct | 0.0182 | (0.0084 , 0.0281) | 0.000455541 | 0.8497(0.7908 - 0.913) | 1.83E-05 |
| S_VLDL_CE_pct | 0.0087 | (-0.0014 , 0.0189) | 0.10453243 | 0.9113(0.8488 - 0.9785) | 0.016053317 |
| S_VLDL_FC_pct | 0.0276 | (0.018 , 0.0373) | 4.19E-08 | 0.7866(0.73 - 0.8477) | 1.05E-09 |
| S_VLDL_TG_pct | -0.0215 | (-0.0313 , -0.0117) | 3.06E-05 | 1.2028(1.1185 - 1.2934) | 1.43E-06 |
| XS_VLDL_PL_pct | -1.00E-04 | (-0.0102 , 0.0099) | 0.9779822 | 1.3495(1.2537 - 1.4527) | 1.51E-14 |
| XS_VLDL_C_pct | 0.024 | (0.0148 , 0.0332) | 7.26E-07 | 0.7604(0.7098 - 0.8146) | 5.61E-14 |
| XS_VLDL_CE_pct | 0.0232 | (0.014 , 0.0324) | 1.48E-06 | 0.7454(0.6949 - 0.7996) | 3.26E-15 |
| XS_VLDL_FC_pct | 0.0229 | (0.0131 , 0.0327) | 8.32E-06 | 0.9015(0.8451 - 0.9618) | 0.002836002 |
| XS_VLDL_TG_pct | -0.0269 | (-0.0361 , -0.0177) | 2.64E-08 | 1.2702(1.1866 - 1.3598) | 3.05E-11 |
| IDL_PL_pct | 0.0349 | (0.0246 , 0.0451) | 7.36E-11 | 1.0816(1.0081 - 1.1605) | 0.041922731 |
| IDL_C_pct | 0.0091 | (-2e-04 , 0.0184) | 0.065930889 | 0.7906(0.7429 - 0.8413) | 8.63E-13 |
| IDL_CE_pct | -0.0038 | (-0.0133 , 0.0057) | 0.459973949 | 0.7998(0.7493 - 0.8537) | 9.58E-11 |
| IDL_FC_pct | 0.0326 | (0.0226 , 0.0425) | 4.12E-10 | 0.852(0.7979 - 0.9099) | 3.79E-06 |
| IDL_TG_pct | -0.0236 | (-0.0326 , -0.0145) | 6.99E-07 | 1.2745(1.2025 - 1.3508) | 4.21E-15 |
| L_LDL_FC_pct | 0.0303 | (0.0212 , 0.0393) | 1.75E-10 | 0.7653(0.7197 - 0.8137) | 2.52E-16 |
| M_LDL_PL_pct | 0.0087 | (-0.0018 , 0.0192) | 0.118516773 | 1.0035(0.9334 - 1.0787) | 0.932932021 |
| M_LDL_CE_pct | -0.0283 | (-0.0386 , -0.0181) | 1.43E-07 | 1.1017(1.0228 - 1.1867) | 0.016108039 |
| M_LDL_FC_pct | 0.0385 | (0.029 , 0.0481) | 1.85E-14 | 0.7709(0.7225 - 0.8226) | 3.45E-14 |
| S_LDL_PL_pct | 0.0387 | (0.0284 , 0.0491) | 8.56E-13 | 0.9539(0.8867 - 1.0262) | 0.242008779 |
| S_LDL_C_pct | -0.0088 | (-0.0185 , 0.001) | 0.090597698 | 0.8536(0.7995 - 0.9112) | 4.45E-06 |
| S_LDL_CE_pct | -0.0389 | (-0.0492 , -0.0286) | 5.88E-13 | 1.0593(0.9857 - 1.1383) | 0.152252178 |
| S_LDL_FC_pct | 0.0316 | (0.022 , 0.0411) | 3.04E-10 | 0.8288(0.782 - 0.8783) | 8.11E-10 |
| XL_HDL_C_pct | -0.0179 | (-0.0276 , -0.0083) | 0.000428466 | 1.029(0.9568 - 1.1068) | 0.487605426 |
| XL_HDL_CE_pct | 0.0107 | (9e-04 , 0.0205) | 0.040252567 | 0.8637(0.8183 - 0.9117) | 2.60E-07 |
| L_HDL_PL_pct | -0.0597 | (-0.0689 , -0.0506) | 9.96E-36 | 1.3065(1.2355 - 1.3816) | 2.27E-19 |
| L_HDL_C_pct | 0.0535 | (0.0443 , 0.0627) | 1.66E-28 | 0.7727(0.7321 - 0.8155) | 2.27E-19 |
| L_HDL_CE_pct | 0.0554 | (0.046 , 0.0647) | 1.94E-29 | 0.7613(0.7202 - 0.8047) | 2.32E-20 |
| M_HDL_PL_pct | -0.0421 | (-0.0517 , -0.0325) | 7.57E-17 | 1.4086(1.3156 - 1.5081) | 5.18E-21 |
| M_HDL_C_pct | 0.0418 | (0.0321 , 0.0515) | 2.77E-16 | 0.7296(0.6804 - 0.7825) | 2.41E-17 |
| M_HDL_CE_pct | 0.0417 | (0.0318 , 0.0516) | 1.30E-15 | 0.7599(0.7104 - 0.8128) | 1.37E-14 |
| M_HDL_FC_pct | 0.0234 | (0.0143 , 0.0325) | 9.27E-07 | 0.7195(0.6639 - 0.7798) | 1.22E-14 |
| M_HDL_TG_pct | -0.0385 | (-0.0483 , -0.0287) | 8.29E-14 | 1.3088(1.2202 - 1.4037) | 3.78E-13 |
| S_HDL_PL_pct | -0.015 | (-0.0252 , -0.0049) | 0.005050989 | 0.991(0.9197 - 1.0678) | 0.825928101 |
| S_HDL_C_pct | 0.0344 | (0.0243 , 0.0446) | 7.98E-11 | 0.8311(0.7764 - 0.8896) | 2.40E-07 |
| S_HDL_CE_pct | 0.024 | (0.0139 , 0.0342) | 6.90E-06 | 0.8634(0.8066 - 0.9243) | 4.86E-05 |
| S_HDL_FC_pct | 0.0369 | (0.0271 , 0.0468) | 9.31E-13 | 0.842(0.7759 - 0.9137) | 7.46E-05 |
| S_HDL_TG_pct | -0.0348 | (-0.0446 , -0.0251) | 7.34E-12 | 1.3404(1.2494 - 1.438) | 4.26E-15 |
| GL | -0.0157 | (-0.0252 , -0.0061) | 0.001919751 | 1.2096(1.1489 - 1.2735) | 2.77E-12 |
| Omega_6_by_Omega_3 | -0.0039 | (-0.014 , 0.0063) | 0.482851146 | 1.0973(1.0196 - 1.1809) | 0.019934255 |
| His | 6.00E-04 | (-0.01 , 0.0111) | 0.924811718 | 0.8914(0.8305 - 0.9568) | 0.002498798 |
| Phe | -0.0258 | (-0.0363 , -0.0152) | 3.05E-06 | 1.1593(1.0789 - 1.2457) | 0.000109254 |
| Glucose | -0.0081 | (-0.0177 , 0.0016) | 0.115116096 | 1.2106(1.1422 - 1.2831) | 4.75E-10 |
| Pyruvate | -0.012 | (-0.0228 , -0.0012) | 0.03560413 | 1.1185(1.0394 - 1.2035) | 0.004448617 |
| bOHbutyrate | -0.0015 | (-0.0123 , 0.0092) | 0.797735884 | 1.0485(0.9749 - 1.1277) | 0.239081052 |
| Acetate | 0.0253 | (0.0155 , 0.0351) | 9.08E-07 | 0.9643(0.8997 - 1.0336) | 0.3494822 |
| Acetoacetate | -0.0143 | (-0.025 , -0.0036) | 0.01128457 | 1.0718(0.995 - 1.1546) | 0.091291831 |
| Acetone | -0.0024 | (-0.0127 , 0.008) | 0.682676153 | 0.9813(0.9121 - 1.0558) | 0.652593406 |
| Creatinine | -0.038 | (-0.0464 , -0.0296) | 6.61E-18 | 1.0601(0.9732 - 1.1548) | 0.21780519 |
| XXL_VLDL_C_pct | 0.0115 | (-3e-04 , 0.0234) | 0.065930889 | 0.8946(0.8276 - 0.967) | 0.007954795 |
| XXL_VLDL_CE_pct | -4.00E-04 | (-0.0122 , 0.0115) | 0.955092148 | 0.9313(0.8635 - 1.0044) | 0.088173763 |
| XXL_VLDL_FC_pct | 0.0166 | (0.0046 , 0.0286) | 0.008963041 | 0.8763(0.8119 - 0.9459) | 0.001251991 |
| XL_VLDL_PL_pct | -0.0179 | (-0.032 , -0.0037) | 0.016925733 | 1.0327(0.9446 - 1.1291) | 0.527624568 |
| XL_VLDL_CE_pct | 0.0375 | (0.0272 , 0.0479) | 3.79E-12 | 0.7856(0.7274 - 0.8485) | 2.49E-09 |
| L_VLDL_PL_pct | -0.0205 | (-0.0311 , -0.0098) | 0.000267049 | 1.0812(0.9626 - 1.2145) | 0.224603047 |
| L_LDL_PL_pct | -0.0102 | (-0.0202 , -1e-04) | 0.056774975 | 1.0156(0.9454 - 1.091) | 0.696457064 |
| L_LDL_C_pct | 0.0062 | (0.0041 , 0.0084) | 4.39E-08 | 0.9015(0.8828 - 0.9207) | 2.08E-20 |
| L_LDL_CE_pct | 0.0029 | (-4e-04 , 0.0061) | 0.09515859 | 0.9335(0.8968 - 0.9717) | 0.001344089 |
| L_LDL_TG_pct | -0.0278 | (-0.0373 , -0.0183) | 2.64E-08 | 1.31(1.2259 - 1.3999) | 1.51E-14 |
| M_LDL_C_pct | 0.0025 | (5e-04 , 0.0045) | 0.016799404 | 0.8412(0.7962 - 0.8888) | 2.33E-09 |
| M_LDL_TG_pct | -0.0181 | (-0.0278 , -0.0084) | 0.000393264 | 1.2428(1.163 - 1.3281) | 5.34E-10 |
| S_LDL_TG_pct | -0.0294 | (-0.0391 , -0.0196) | 8.40E-09 | 1.2609(1.1771 - 1.3506) | 1.76E-10 |
| XL_HDL_PL_pct | 0.0154 | (0.0059 , 0.025) | 0.002264983 | 0.9661(0.9216 - 1.0127) | 0.184692534 |
| XL_HDL_FC_pct | -0.037 | (-0.046 , -0.028) | 6.96E-15 | 1.3118(1.2239 - 1.406) | 1.32E-13 |
| XL_HDL_TG_pct | -0.0492 | (-0.0588 , -0.0396) | 1.71E-22 | 1.3055(1.2158 - 1.4018) | 1.39E-12 |
| L_HDL_FC_pct | 0.012 | (0.0033 , 0.0207) | 0.009417455 | 0.964(0.9311 - 0.998) | 0.054568199 |
| L_HDL_TG_pct | -0.0444 | (-0.0543 , -0.0345) | 9.62E-18 | 1.3111(1.2159 - 1.4136) | 1.08E-11 |

| **Supplementary Table 5.** The criteria for the frailty phenotype in the UK Biobank. | | |
| --- | --- | --- |
| **Frailty indicators** | **Question description** | **Response** |
| **Exhaustion** | “Over the past 2 weeks, how often have you felt tired or had little energy?” | 1: “More than half the days or nearly every day”; 0: Others |
| **Low physical activity** | “In the last 4 weeks, did you spend any time doing light DIYa activity, heavy DIY activity, or strenuous sports?” | 1: “None or light activity with a frequency of once per week or less”; 0: Others |
| **Slow Walking Speed** | “How would you describe your usual walking pace?” | 1: “Slow pace”; 0: Others |
| **Weak Hand Grip** | Grip strength was measured using the Jamar J00105 hydraulic hand dynamometer (Lafayette Instrument). Participants were asked to complete a grip assessment for both hands once. The maximal value of the right and left hands was used | 1: Males:  grip strength ≤29 kg and BMI ≤24 kg/m^2^;  grip strength ≤30 kg and BMI 24.1-28 kg/m^2^;  grip strength ≤32 kg and BMI >28 kg/m2;  Females:  grip strength ≤17 kg and BMI ≤23 kg/m^2^;  grip strength ≤17.3 kg and BMI 23.1-26 kg/m^2^;  grip strength ≤18 kg and BMI 26.1-29 kg/m^2^; grip strength ≤21 kg and BMI >29 kg/m^2^;  0: Others |
| **Weight Loss** | “Compared with one year ago, has your weight changed?” | 1: “Yes, loss weight”; 0: Others |

| **Supplementary Table 6.** EAT-Lancet diet score criteria constructed for the assessment of the EAT-Lancet diet and examples of food in the UK Biobank^1,2^ | | |
| --- | --- | --- |
| **Component** | **Criteria for 0 points** | **Criteria for 1 points** |
| **Whole grains** |  |  |
| 1 Rice, wheat, corn, and other | >464 g/day | ≤464 g/day |
| **Tubers and starchy begetables** |  |  |
| 2 Potatoes | >100 g/day | ≤100 g/day |
| **Vegetables** |  |  |
| 3 All vegetables | <200 g/day | ≥200 g/day |
| **Fruits** |  |  |
| 4 All fruits | <100 g/day | ≥100 g/day |
| **Dairy foods** |  |  |
| 5 Whole milk or derivative equivalents | >500 g/day | ≤500 g/day |
| **Protein sources** |  |  |
| 6 Beef, lamb, pork | >28 g/day | ≤28 g/day |
| 7 Chicken, other poultry | >58 g/day | ≤58 g/day |
| 8 Eggs | >25 g/day | ≤25 g/day |
| 9 Fish | >100 g/day | ≤100 g/day |
| 10 Dry beans, lentils, peas | >100 g/day | ≤100 g/day |
| 11 Soy foods | >50 g/day | ≤50 g/day |
| 12 Peanuts or tree nuts | <25 g/day | ≥25 g/day |
| **Added fats** |  |  |
| 13 Palm oil, unsaturated oils, dairy fats (incl. in milk), lard or tallow |  | Ratio of 0.8 for unsaturated: saturated fat intake |
| **Add sugar** |  |  |
| 14 All sweeteners | >31 g/day | ≤31 g/day |

| **Supplementary Table 7.** Detailed information on missing covariates. | |
| --- | --- |
| Variable | The proportion of missing values |
| Age | 0.00% |
| Sex | 0.00% |
| Energy | 76.50% |
| Physical activity | 12.93% |
| Townsend Deprivation index | 0.10% |
| Education | 0.22% |
| Employment | 0.56% |
| Smoking | 0.18% |
| Drinking | 0.02% |
| Cardiovascular disease | 0.00% |
| Cancer | 0.00% |

| **Supplementary Table 8.** Metabolite categories based on the UK Biobank | | | |
| --- | --- | --- | --- |
| **Metabolite** | **Shortened name** | **Units** | **Group** |
| Total Cholesterol | Total_C | mmol/L | Cholesterol |
| Total Cholesterol Minus HDL-C | non_HDL_C | mmol/L | Cholesterol |
| Remnant Cholesterol (Non-HDL, Non-LDL -Cholesterol) | Remnant_C | mmol/L | Cholesterol |
| VLDL Cholesterol | VLDL_C | mmol/L | Cholesterol |
| Clinical LDL Cholesterol | Clinical_LDL_C | mmol/L | Cholesterol |
| LDL Cholesterol | LDL_C | mmol/L | Cholesterol |
| HDL Cholesterol | HDL_C | mmol/L | Cholesterol |
| Total Triglycerides | Total_TG | mmol/L | Triglycerides |
| Triglycerides in VLDL | VLDL_TG | mmol/L | Triglycerides |
| Triglycerides in LDL | LDL_TG | mmol/L | Triglycerides |
| Triglycerides in HDL | HDL_TG | mmol/L | Triglycerides |
| Total Phospholipids in Lipoprotein Particles | Total_PL | mmol/L | Phospholipids |
| Phospholipids in VLDL | VLDL_PL | mmol/L | Phospholipids |
| Phospholipids in LDL | LDL_PL | mmol/L | Phospholipids |
| Phospholipids in HDL | HDL_PL | mmol/L | Phospholipids |
| Total Esterified Cholesterol | Total_CE | mmol/L | Cholesteryl esters |
| Cholesteryl Esters in VLDL | VLDL_CE | mmol/L | Cholesteryl esters |
| Cholesteryl Esters in LDL | LDL_CE | mmol/L | Cholesteryl esters |
| Cholesteryl Esters in HDL | HDL_CE | mmol/L | Cholesteryl esters |
| Total Free Cholesterol | Total_FC | mmol/L | Free cholesterol |
| Free Cholesterol in VLDL | VLDL_FC | mmol/L | Free cholesterol |
| Free Cholesterol in LDL | LDL_FC | mmol/L | Free cholesterol |
| Free Cholesterol in HDL | HDL_FC | mmol/L | Free cholesterol |
| Total Lipids in Lipoprotein Particles | Total_L | mmol/L | Total lipids |
| Total Lipids in VLDL | VLDL_L | mmol/L | Total lipids |
| Total Lipids in LDL | LDL_L | mmol/L | Total lipids |
| Total Lipids in HDL | HDL_L | mmol/L | Total lipids |
| Total Concentration of Lipoprotein Particles | Total_P | mmol/L | Lipoprotein particle concentrations |
| Concentration of VLDL Particles | VLDL_P | mmol/L | Lipoprotein particle concentrations |
| Concentration of LDL Particles | LDL_P | mmol/L | Lipoprotein particle concentrations |
| Concentration of HDL Particles | HDL_P | mmol/L | Lipoprotein particle concentrations |
| Average Diameter for VLDL Particles | VLDL_size | nm | Lipoprotein particle sizes |
| Average Diameter for LDL Particles | LDL_size | nm | Lipoprotein particle sizes |
| Average Diameter for HDL Particles | HDL_size | nm | Lipoprotein particle sizes |
| Phosphoglycerides | Phosphoglyc | mmol/L | Other lipids |
| Triglycerides to Phosphoglycerides ratio | TG_by_PG | ratio | Other lipids |
| Total Cholines | Cholines | mmol/L | Other lipids |
| Phosphatidylcholines | Phosphatidylc | mmol/L | Other lipids |
| Sphingomyelins | Sphingomyelins | mmol/L | Other lipids |
| Apolipoprotein B | ApoB | g/l | Apolipoproteins |
| Apolipoprotein A1 | ApoA1 | g/l | Apolipoproteins |
| Apolipoprotein B to Apolipoprotein A1 ratio | ApoB_by_ApoA1 | ratio | Apolipoproteins |
| Total Fatty Acids | Total_FA | mmol/L | Fatty acids |
| Degree of Unsaturation | Unsaturation | degree | Fatty acids |
| Omega-3 Fatty Acids | Omega_3 | mmol/L | Fatty acids |
| Omega-6 Fatty Acids | Omega_6 | mmol/L | Fatty acids |
| Polyunsaturated Fatty Acids | PUFA | mmol/L | Fatty acids |
| Monounsaturated Fatty Acids | MUFA | mmol/L | Fatty acids |
| Saturated Fatty Acids | SFA | mmol/L | Fatty acids |
| Linoleic Acid | LA | mmol/L | Fatty acids |
| Docosahexaenoic Acid | DHA | mmol/L | Fatty acids |
| Omega-3 Fatty Acids to Total Fatty Acids percentage | Omega_3_pct | % | Fatty acids |
| Omega-6 Fatty Acids to Total Fatty Acids percentage | Omega_6_pct | % | Fatty acids |
| Polyunsaturated Fatty Acids to Total Fatty Acids percentage | PUFA_pct | % | Fatty acids |
| Monounsaturated Fatty Acids to Total Fatty Acids percentage | MUFA_pct | % | Fatty acids |
| Saturated Fatty Acids to Total Fatty Acids percentage | SFA_pct | % | Fatty acids |
| Linoleic Acid to Total Fatty Acids percentage | LA_pct | % | Fatty acids |
| Docosahexaenoic Acid to Total Fatty Acids percentage | DHA_pct | % | Fatty acids |
| Polyunsaturated Fatty Acids to Monounsaturated Fatty Acids ratio | PUFA_by_MUFA | ratio | Fatty acids |
| Omega-6 Fatty Acids to Omega-3 Fatty Acids ratio | Omega_6_by_Omega_3 | ratio | Fatty acids |
| Alanine | Ala | mmol/L | Amino acids |
| Glutamine | Gln | mmol/L | Amino acids |
| Glycine | Gly | mmol/L | Amino acids |
| Histidine | His | mmol/L | Amino acids |
| Total Concentration of Branched-Chain Amino Acids (Leucine + Isoleucine + Valine) | Total_BCAA | mmol/L | Amino acids |
| Isoleucine | Ile | mmol/L | Amino acids |
| Leucine | Leu | mmol/L | Amino acids |
| Valine | Val | mmol/L | Amino acids |
| Phenylalanine | Phe | mmol/L | Amino acids |
| Tyrosine | Tyr | mmol/L | Amino acids |
| Glucose | Glucose | mmol/L | Glycolysis related metabolites |
| Lactate | Lactate | mmol/L | Glycolysis related metabolites |
| Pyruvate | Pyruvate | mmol/L | Glycolysis related metabolites |
| Citrate | Citrate | mmol/L | Glycolysis related metabolites |
| 3-Hydroxybutyrate | bOHbutyrate | mmol/L | Ketone bodies |
| Acetate | Acetate | mmol/L | Ketone bodies |
| Acetoacetate | Acetoacetate | mmol/L | Ketone bodies |
| Acetone | Acetone | mmol/L | Ketone bodies |
| Creatinine | Creatinine | mmol/L | Fluid balance |
| Albumin | Albumin | g/l | Fluid balance |
| Glycoprotein Acetyls | GlycA | mmol/L | Inflammation |
| Concentration of Chylomicrons and Extremely Large VLDL Particles | XXL_VLDL_P | mmol/L | Lipoprotein subclasses |
| Total Lipids in Chylomicrons and Extremely Large VLDL | XXL_VLDL_L | mmol/L | Lipoprotein subclasses |
| Phospholipids in Chylomicrons and Extremely Large VLDL | XXL_VLDL_PL | mmol/L | Lipoprotein subclasses |
| Cholesterol in Chylomicrons and Extremely Large VLDL | XXL_VLDL_C | mmol/L | Lipoprotein subclasses |
| Cholesteryl Esters in Chylomicrons and Extremely Large VLDL | XXL_VLDL_CE | mmol/L | Lipoprotein subclasses |
| Free Cholesterol in Chylomicrons and Extremely Large VLDL | XXL_VLDL_FC | mmol/L | Lipoprotein subclasses |
| Triglycerides in Chylomicrons and Extremely Large VLDL | XXL_VLDL_TG | mmol/L | Lipoprotein subclasses |
| Concentration of Very Large VLDL Particles | XL_VLDL_P | mmol/L | Lipoprotein subclasses |
| Total Lipids in Very Large VLDL | XL_VLDL_L | mmol/L | Lipoprotein subclasses |
| Phospholipids in Very Large VLDL | XL_VLDL_PL | mmol/L | Lipoprotein subclasses |
| Cholesterol in Very Large VLDL | XL_VLDL_C | mmol/L | Lipoprotein subclasses |
| Cholesteryl Esters in Very Large VLDL | XL_VLDL_CE | mmol/L | Lipoprotein subclasses |
| Free Cholesterol in Very Large VLDL | XL_VLDL_FC | mmol/L | Lipoprotein subclasses |
| Triglycerides in Very Large VLDL | XL_VLDL_TG | mmol/L | Lipoprotein subclasses |
| Concentration of Large VLDL Particles | L_VLDL_P | mmol/L | Lipoprotein subclasses |
| Total Lipids in Large VLDL | L_VLDL_L | mmol/L | Lipoprotein subclasses |
| Phospholipids in Large VLDL | L_VLDL_PL | mmol/L | Lipoprotein subclasses |
| Cholesterol in Large VLDL | L_VLDL_C | mmol/L | Lipoprotein subclasses |
| Cholesteryl Esters in Large VLDL | L_VLDL_CE | mmol/L | Lipoprotein subclasses |
| Free Cholesterol in Large VLDL | L_VLDL_FC | mmol/L | Lipoprotein subclasses |
| Triglycerides in Large VLDL | L_VLDL_TG | mmol/L | Lipoprotein subclasses |
| Concentration of Medium VLDL Particles | M_VLDL_P | mmol/L | Lipoprotein subclasses |
| Total Lipids in Medium VLDL | M_VLDL_L | mmol/L | Lipoprotein subclasses |
| Phospholipids in Medium VLDL | M_VLDL_PL | mmol/L | Lipoprotein subclasses |
| Cholesterol in Medium VLDL | M_VLDL_C | mmol/L | Lipoprotein subclasses |
| Cholesteryl Esters in Medium VLDL | M_VLDL_CE | mmol/L | Lipoprotein subclasses |
| Free Cholesterol in Medium VLDL | M_VLDL_FC | mmol/L | Lipoprotein subclasses |
| Triglycerides in Medium VLDL | M_VLDL_TG | mmol/L | Lipoprotein subclasses |
| Concentration of Small VLDL Particles | S_VLDL_P | mmol/L | Lipoprotein subclasses |
| Total Lipids in Small VLDL | S_VLDL_L | mmol/L | Lipoprotein subclasses |
| Phospholipids in Small VLDL | S_VLDL_PL | mmol/L | Lipoprotein subclasses |
| Cholesterol in Small VLDL | S_VLDL_C | mmol/L | Lipoprotein subclasses |
| Cholesteryl Esters in Small VLDL | S_VLDL_CE | mmol/L | Lipoprotein subclasses |
| Free Cholesterol in Small VLDL | S_VLDL_FC | mmol/L | Lipoprotein subclasses |
| Triglycerides in Small VLDL | S_VLDL_TG | mmol/L | Lipoprotein subclasses |
| Concentration of Very Small VLDL Particles | XS_VLDL_P | mmol/L | Lipoprotein subclasses |
| Total Lipids in Very Small VLDL | XS_VLDL_L | mmol/L | Lipoprotein subclasses |
| Phospholipids in Very Small VLDL | XS_VLDL_PL | mmol/L | Lipoprotein subclasses |
| Cholesterol in Very Small VLDL | XS_VLDL_C | mmol/L | Lipoprotein subclasses |
| Cholesteryl Esters in Very Small VLDL | XS_VLDL_CE | mmol/L | Lipoprotein subclasses |
| Free Cholesterol in Very Small VLDL | XS_VLDL_FC | mmol/L | Lipoprotein subclasses |
| Triglycerides in Very Small VLDL | XS_VLDL_TG | mmol/L | Lipoprotein subclasses |
| Concentration of IDL Particles | IDL_P | mmol/L | Lipoprotein subclasses |
| Total Lipids in IDL | IDL_L | mmol/L | Lipoprotein subclasses |
| Phospholipids in IDL | IDL_PL | mmol/L | Lipoprotein subclasses |
| Cholesterol in IDL | IDL_C | mmol/L | Lipoprotein subclasses |
| Cholesteryl Esters in IDL | IDL_CE | mmol/L | Lipoprotein subclasses |
| Free Cholesterol in IDL | IDL_FC | mmol/L | Lipoprotein subclasses |
| Triglycerides in IDL | IDL_TG | mmol/L | Lipoprotein subclasses |
| Concentration of Large LDL Particles | L_LDL_P | mmol/L | Lipoprotein subclasses |
| Total Lipids in Large LDL | L_LDL_L | mmol/L | Lipoprotein subclasses |
| Phospholipids in Large LDL | L_LDL_PL | mmol/L | Lipoprotein subclasses |
| Cholesterol in Large LDL | L_LDL_C | mmol/L | Lipoprotein subclasses |
| Cholesteryl Esters in Large LDL | L_LDL_CE | mmol/L | Lipoprotein subclasses |
| Free Cholesterol in Large LDL | L_LDL_FC | mmol/L | Lipoprotein subclasses |
| Triglycerides in Large LDL | L_LDL_TG | mmol/L | Lipoprotein subclasses |
| Concentration of Medium LDL Particles | M_LDL_P | mmol/L | Lipoprotein subclasses |
| Total Lipids in Medium LDL | M_LDL_L | mmol/L | Lipoprotein subclasses |
| Phospholipids in Medium LDL | M_LDL_PL | mmol/L | Lipoprotein subclasses |
| Cholesterol in Medium LDL | M_LDL_C | mmol/L | Lipoprotein subclasses |
| Cholesteryl Esters in Medium LDL | M_LDL_CE | mmol/L | Lipoprotein subclasses |
| Free Cholesterol in Medium LDL | M_LDL_FC | mmol/L | Lipoprotein subclasses |
| Triglycerides in Medium LDL | M_LDL_TG | mmol/L | Lipoprotein subclasses |
| Concentration of Small LDL Particles | S_LDL_P | mmol/L | Lipoprotein subclasses |
| Total Lipids in Small LDL | S_LDL_L | mmol/L | Lipoprotein subclasses |
| Phospholipids in Small LDL | S_LDL_PL | mmol/L | Lipoprotein subclasses |
| Cholesterol in Small LDL | S_LDL_C | mmol/L | Lipoprotein subclasses |
| Cholesteryl Esters in Small LDL | S_LDL_CE | mmol/L | Lipoprotein subclasses |
| Free Cholesterol in Small LDL | S_LDL_FC | mmol/L | Lipoprotein subclasses |
| Triglycerides in Small LDL | S_LDL_TG | mmol/L | Lipoprotein subclasses |
| Concentration of Very Large HDL Particles | XL_HDL_P | mmol/L | Lipoprotein subclasses |
| Total Lipids in Very Large HDL | XL_HDL_L | mmol/L | Lipoprotein subclasses |
| Phospholipids in Very Large HDL | XL_HDL_PL | mmol/L | Lipoprotein subclasses |
| Cholesterol in Very Large HDL | XL_HDL_C | mmol/L | Lipoprotein subclasses |
| Cholesteryl Esters in Very Large HDL | XL_HDL_CE | mmol/L | Lipoprotein subclasses |
| Free Cholesterol in Very Large HDL | XL_HDL_FC | mmol/L | Lipoprotein subclasses |
| Triglycerides in Very Large HDL | XL_HDL_TG | mmol/L | Lipoprotein subclasses |
| Concentration of Large HDL Particles | L_HDL_P | mmol/L | Lipoprotein subclasses |
| Total Lipids in Large HDL | L_HDL_L | mmol/L | Lipoprotein subclasses |
| Phospholipids in Large HDL | L_HDL_PL | mmol/L | Lipoprotein subclasses |
| Cholesterol in Large HDL | L_HDL_C | mmol/L | Lipoprotein subclasses |
| Cholesteryl Esters in Large HDL | L_HDL_CE | mmol/L | Lipoprotein subclasses |
| Free Cholesterol in Large HDL | L_HDL_FC | mmol/L | Lipoprotein subclasses |
| Triglycerides in Large HDL | L_HDL_TG | mmol/L | Lipoprotein subclasses |
| Concentration of Medium HDL Particles | M_HDL_P | mmol/L | Lipoprotein subclasses |
| Total Lipids in Medium HDL | M_HDL_L | mmol/L | Lipoprotein subclasses |
| Phospholipids in Medium HDL | M_HDL_PL | mmol/L | Lipoprotein subclasses |
| Cholesterol in Medium HDL | M_HDL_C | mmol/L | Lipoprotein subclasses |
| Cholesteryl Esters in Medium HDL | M_HDL_CE | mmol/L | Lipoprotein subclasses |
| Free Cholesterol in Medium HDL | M_HDL_FC | mmol/L | Lipoprotein subclasses |
| Triglycerides in Medium HDL | M_HDL_TG | mmol/L | Lipoprotein subclasses |
| Concentration of Small HDL Particles | S_HDL_P | mmol/L | Lipoprotein subclasses |
| Total Lipids in Small HDL | S_HDL_L | mmol/L | Lipoprotein subclasses |
| Phospholipids in Small HDL | S_HDL_PL | mmol/L | Lipoprotein subclasses |
| Cholesterol in Small HDL | S_HDL_C | mmol/L | Lipoprotein subclasses |
| Cholesteryl Esters in Small HDL | S_HDL_CE | mmol/L | Lipoprotein subclasses |
| Free Cholesterol in Small HDL | S_HDL_FC | mmol/L | Lipoprotein subclasses |
| Triglycerides in Small HDL | S_HDL_TG | mmol/L | Lipoprotein subclasses |
| Phospholipids to Total Lipids in Chylomicrons and Extremely Large VLDL percentage | XXL_VLDL_PL_pct | % | Relative lipoprotein lipid concentrations |
| Cholesterol to Total Lipids in Chylomicrons and Extremely Large VLDL percentage | XXL_VLDL_C_pct | % | Relative lipoprotein lipid concentrations |
| Cholesteryl Esters to Total Lipids in Chylomicrons and Extremely Large VLDL percentage | XXL_VLDL_CE_pct | % | Relative lipoprotein lipid concentrations |
| Free Cholesterol to Total Lipids in Chylomicrons and Extremely Large VLDL percentage | XXL_VLDL_FC_pct | % | Relative lipoprotein lipid concentrations |
| Triglycerides to Total Lipids in Chylomicrons and Extremely Large VLDL percentage | XXL_VLDL_TG_pct | % | Relative lipoprotein lipid concentrations |
| Phospholipids to Total Lipids in Very Large VLDL percentage | XL_VLDL_PL_pct | % | Relative lipoprotein lipid concentrations |
| Cholesterol to Total Lipids in Very Large VLDL percentage | XL_VLDL_C_pct | % | Relative lipoprotein lipid concentrations |
| Cholesteryl Esters to Total Lipids in Very Large VLDL percentage | XL_VLDL_CE_pct | % | Relative lipoprotein lipid concentrations |
| Free Cholesterol to Total Lipids in Very Large VLDL percentage | XL_VLDL_FC_pct | % | Relative lipoprotein lipid concentrations |
| Triglycerides to Total Lipids in Very Large VLDL percentage | XL_VLDL_TG_pct | % | Relative lipoprotein lipid concentrations |
| Phospholipids to Total Lipids in Large VLDL percentage | L_VLDL_PL_pct | % | Relative lipoprotein lipid concentrations |
| Cholesterol to Total Lipids in Large VLDL percentage | L_VLDL_C_pct | % | Relative lipoprotein lipid concentrations |
| Cholesteryl Esters to Total Lipids in Large VLDL percentage | L_VLDL_CE_pct | % | Relative lipoprotein lipid concentrations |
| Free Cholesterol to Total Lipids in Large VLDL percentage | L_VLDL_FC_pct | % | Relative lipoprotein lipid concentrations |
| Triglycerides to Total Lipids in Large VLDL percentage | L_VLDL_TG_pct | % | Relative lipoprotein lipid concentrations |
| Phospholipids to Total Lipids in Medium VLDL percentage | M_VLDL_PL_pct | % | Relative lipoprotein lipid concentrations |
| Cholesterol to Total Lipids in Medium VLDL percentage | M_VLDL_C_pct | % | Relative lipoprotein lipid concentrations |
| Cholesteryl Esters to Total Lipids in Medium VLDL percentage | M_VLDL_CE_pct | % | Relative lipoprotein lipid concentrations |
| Free Cholesterol to Total Lipids in Medium VLDL percentage | M_VLDL_FC_pct | % | Relative lipoprotein lipid concentrations |
| Triglycerides to Total Lipids in Medium VLDL percentage | M_VLDL_TG_pct | % | Relative lipoprotein lipid concentrations |
| Phospholipids to Total Lipids in Small VLDL percentage | S_VLDL_PL_pct | % | Relative lipoprotein lipid concentrations |
| Cholesterol to Total Lipids in Small VLDL percentage | S_VLDL_C_pct | % | Relative lipoprotein lipid concentrations |
| Cholesteryl Esters to Total Lipids in Small VLDL percentage | S_VLDL_CE_pct | % | Relative lipoprotein lipid concentrations |
| Free Cholesterol to Total Lipids in Small VLDL percentage | S_VLDL_FC_pct | % | Relative lipoprotein lipid concentrations |
| Triglycerides to Total Lipids in Small VLDL percentage | S_VLDL_TG_pct | % | Relative lipoprotein lipid concentrations |
| Phospholipids to Total Lipids in Very Small VLDL percentage | XS_VLDL_PL_pct | % | Relative lipoprotein lipid concentrations |
| Cholesterol to Total Lipids in Very Small VLDL percentage | XS_VLDL_C_pct | % | Relative lipoprotein lipid concentrations |
| Cholesteryl Esters to Total Lipids in Very Small VLDL percentage | XS_VLDL_CE_pct | % | Relative lipoprotein lipid concentrations |
| Free Cholesterol to Total Lipids in Very Small VLDL percentage | XS_VLDL_FC_pct | % | Relative lipoprotein lipid concentrations |
| Triglycerides to Total Lipids in Very Small VLDL percentage | XS_VLDL_TG_pct | % | Relative lipoprotein lipid concentrations |
| Phospholipids to Total Lipids in IDL percentage | IDL_PL_pct | % | Relative lipoprotein lipid concentrations |
| Cholesterol to Total Lipids in IDL percentage | IDL_C_pct | % | Relative lipoprotein lipid concentrations |
| Cholesteryl Esters to Total Lipids in IDL percentage | IDL_CE_pct | % | Relative lipoprotein lipid concentrations |
| Free Cholesterol to Total Lipids in IDL percentage | IDL_FC_pct | % | Relative lipoprotein lipid concentrations |
| Triglycerides to Total Lipids in IDL percentage | IDL_TG_pct | % | Relative lipoprotein lipid concentrations |
| Phospholipids to Total Lipids in Large LDL percentage | L_LDL_PL_pct | % | Relative lipoprotein lipid concentrations |
| Cholesterol to Total Lipids in Large LDL percentage | L_LDL_C_pct | % | Relative lipoprotein lipid concentrations |
| Cholesteryl Esters to Total Lipids in Large LDL percentage | L_LDL_CE_pct | % | Relative lipoprotein lipid concentrations |
| Free Cholesterol to Total Lipids in Large LDL percentage | L_LDL_FC_pct | % | Relative lipoprotein lipid concentrations |
| Triglycerides to Total Lipids in Large LDL percentage | L_LDL_TG_pct | % | Relative lipoprotein lipid concentrations |
| Phospholipids to Total Lipids in Medium LDL percentage | M_LDL_PL_pct | % | Relative lipoprotein lipid concentrations |
| Cholesterol to Total Lipids in Medium LDL percentage | M_LDL_C_pct | % | Relative lipoprotein lipid concentrations |
| Cholesteryl Esters to Total Lipids in Medium LDL percentage | M_LDL_CE_pct | % | Relative lipoprotein lipid concentrations |
| Free Cholesterol to Total Lipids in Medium LDL percentage | M_LDL_FC_pct | % | Relative lipoprotein lipid concentrations |
| Triglycerides to Total Lipids in Medium LDL percentage | M_LDL_TG_pct | % | Relative lipoprotein lipid concentrations |
| Phospholipids to Total Lipids in Small LDL percentage | S_LDL_PL_pct | % | Relative lipoprotein lipid concentrations |
| Cholesterol to Total Lipids in Small LDL percentage | S_LDL_C_pct | % | Relative lipoprotein lipid concentrations |
| Cholesteryl Esters to Total Lipids in Small LDL percentage | S_LDL_CE_pct | % | Relative lipoprotein lipid concentrations |
| Free Cholesterol to Total Lipids in Small LDL percentage | S_LDL_FC_pct | % | Relative lipoprotein lipid concentrations |
| Triglycerides to Total Lipids in Small LDL percentage | S_LDL_TG_pct | % | Relative lipoprotein lipid concentrations |
| Phospholipids to Total Lipids in Very Large HDL percentage | XL_HDL_PL_pct | % | Relative lipoprotein lipid concentrations |
| Cholesterol to Total Lipids in Very Large HDL percentage | XL_HDL_C_pct | % | Relative lipoprotein lipid concentrations |
| Cholesteryl Esters to Total Lipids in Very Large HDL percentage | XL_HDL_CE_pct | % | Relative lipoprotein lipid concentrations |
| Free Cholesterol to Total Lipids in Very Large HDL percentage | XL_HDL_FC_pct | % | Relative lipoprotein lipid concentrations |
| Triglycerides to Total Lipids in Very Large HDL percentage | XL_HDL_TG_pct | % | Relative lipoprotein lipid concentrations |
| Phospholipids to Total Lipids in Large HDL percentage | L_HDL_PL_pct | % | Relative lipoprotein lipid concentrations |
| Cholesterol to Total Lipids in Large HDL percentage | L_HDL_C_pct | % | Relative lipoprotein lipid concentrations |
| Cholesteryl Esters to Total Lipids in Large HDL percentage | L_HDL_CE_pct | % | Relative lipoprotein lipid concentrations |
| Free Cholesterol to Total Lipids in Large HDL percentage | L_HDL_FC_pct | % | Relative lipoprotein lipid concentrations |
| Triglycerides to Total Lipids in Large HDL percentage | L_HDL_TG_pct | % | Relative lipoprotein lipid concentrations |
| Phospholipids to Total Lipids in Medium HDL percentage | M_HDL_PL_pct | % | Relative lipoprotein lipid concentrations |
| Cholesterol to Total Lipids in Medium HDL percentage | M_HDL_C_pct | % | Relative lipoprotein lipid concentrations |
| Cholesteryl Esters to Total Lipids in Medium HDL percentage | M_HDL_CE_pct | % | Relative lipoprotein lipid concentrations |
| Free Cholesterol to Total Lipids in Medium HDL percentage | M_HDL_FC_pct | % | Relative lipoprotein lipid concentrations |
| Triglycerides to Total Lipids in Medium HDL percentage | M_HDL_TG_pct | % | Relative lipoprotein lipid concentrations |
| Phospholipids to Total Lipids in Small HDL percentage | S_HDL_PL_pct | % | Relative lipoprotein lipid concentrations |
| Cholesterol to Total Lipids in Small HDL percentage | S_HDL_C_pct | % | Relative lipoprotein lipid concentrations |
| Cholesteryl Esters to Total Lipids in Small HDL percentage | S_HDL_CE_pct | % | Relative lipoprotein lipid concentrations |
| Free Cholesterol to Total Lipids in Small HDL percentage | S_HDL_FC_pct | % | Relative lipoprotein lipid concentrations |
| Triglycerides to Total Lipids in Small HDL percentage | S_HDL_TG_pct | % | Relative lipoprotein lipid concentrations |
| Glucose-lactate | GL | mmol/L | Glycolysis related metabolites |
| Spectrometer-corrected alanine | SCA | mmol/L | Amino acids |

**
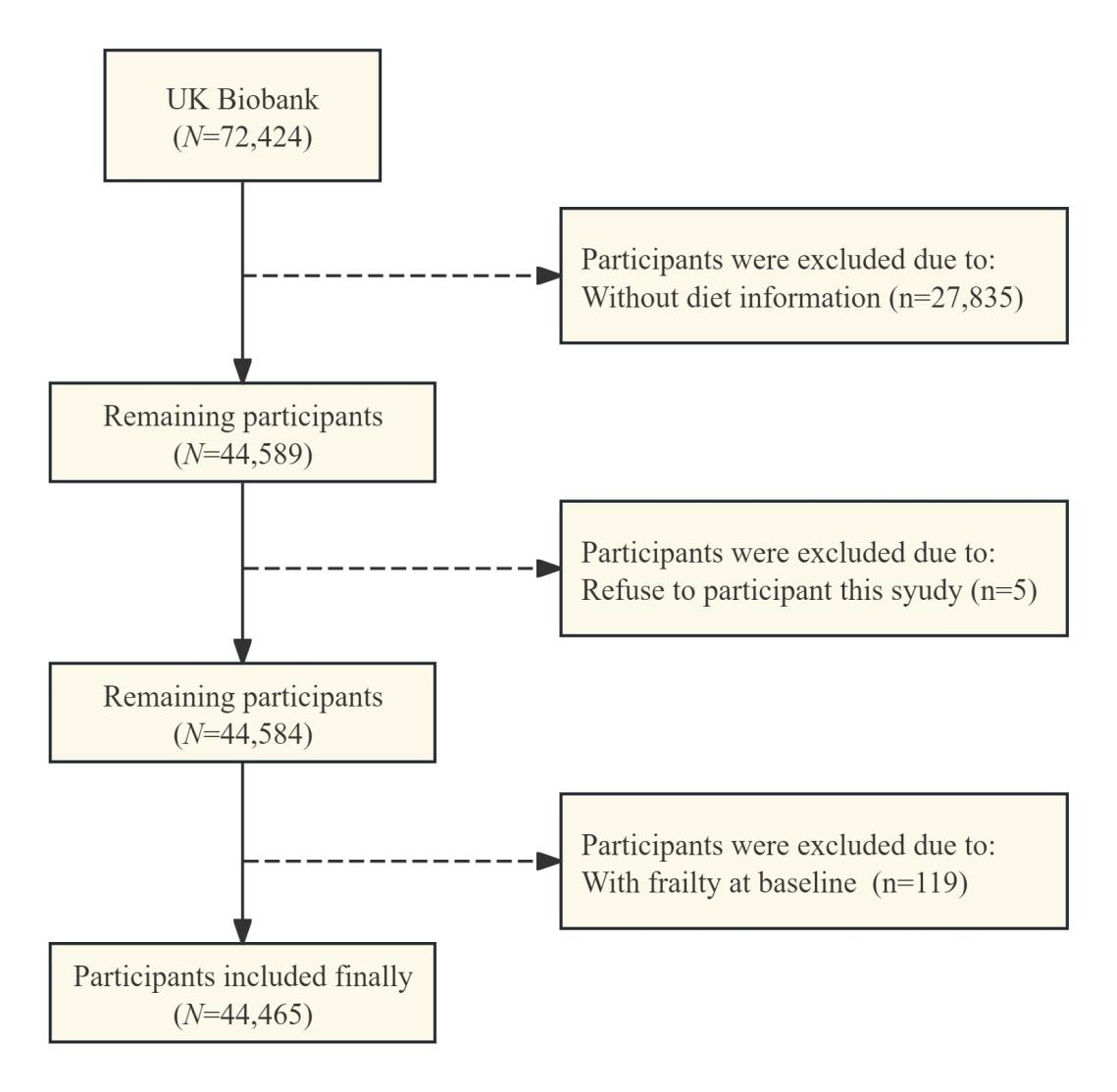
**

**Supplementary Figure 1.** Flowchart of the study.

**Reference**

1. Knuppel A, Papier K, Key TJ, Travis RC. EAT-Lancet score and major health outcomes: the EPIC-Oxford study. Lancet. 2019;394(10194):213-214. doi:10.1016/S0140-6736(19)31236-X
2. Lu X, Wu L, Shao L, et al. Adherence to the EAT-Lancet diet and incident depression and anxiety. Nat Commun. 2024;15(1):5599. Published 2024 Jul 3. doi:10.1038/s41467-024-49653-8
